# The pressure response of Jahn–Teller-distorted Prussian blue analogues†

**DOI:** 10.1039/d3sc06912e

**Published:** 2024-01-23

**Authors:** Hanna L. B. Boström, Andrew B. Cairns, Muzi Chen, Dominik Daisenberger, Christopher J. Ridley, Nicholas P. Funnell

**Affiliations:** a Max Planck Institute for Solid State Research Heisenbergstraße 1 D-70569 Stuttgart Germany; b Department of Materials and Environmental Chemistry, Stockholm University Svante Arrhenius väg 16C SE-106 91 Stockholm Sweden hanna.bostrom@mmk.su.se; c Wallenberg Initiative Materials Science for Sustainability, Department of Materials and Environmental Chemistry, Stockholm University SE-114 18 Stockholm Sweden; d Department of Materials, Imperial College London, Royal School of Mines Exhibition Road SW7 2AZ London UK; e London Centre for Nanotechnology, Imperial College London SW7 2AZ London UK; f Diamond Light Source Ltd, Harwell Campus Didcot OX11 0DE UK; g ISIS Neutron and Muon Source, Rutherford Appleton Laboratory Harwell Campus Didcot OX11 0QX UK

## Abstract

Jahn–Teller (JT) distorted Cu^II^-containing compounds often display interesting structural and functional behaviour upon compression. We use high-pressure X-ray and neutron diffraction to investigate four JT-distorted Prussian blue analogues: Cu[Co(CN)_6_]_0.67_, CuPt(CN)_6_, and ACuCo(CN)_6_ (A = Rb, Cs), where the first two were studied in both their hydrated and dehydrated forms. All compounds are less compressible than the JT-inactive Mn^II^-based counterparts, indicating a coupling between the electronic and mechanical properties. The effect is particularly strong for Cu[Co(CN)_6_]_0.67_, where the local JT distortions are uncorrelated (so-called orbital disorder). This sample amorphises at 0.5 GPa when dehydrated. CuPt(CN)_6_ behaves similarly to the Mn^II^-analogues, with phase transitions at around 1 GPa and low sensitivity to water. For ACuCo(CN)_6_, the JT distortions reduce the propensity for phase transitions, although RbCuCo(CN)_6_ transitions to a new phase (*P*2/*m*) around 3 GPa. Our results have a bearing on both the topical Prussian blue analogues and the wider field of flexible frameworks.

## Abstract 2

Jahn–Teller (JT) distorted Cu^II^-containing compounds often display interesting structural and functional behaviour upon compression.

## Graphical abstract

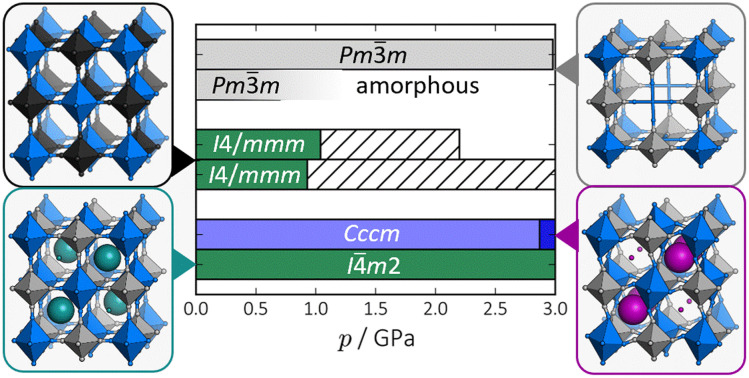

## 1 Introduction

Jahn–Teller (JT) distortions play a central role in solid-state chemistry, and are implicated in *e.g.* the superconductivity of cuprates,^1^ and the colossal magnetoresistance in perovskite manganites.^2^ Studies under pressure have been a useful tool to improve the understanding of the magnetic and electronic effects, and the ability to tune these.^3^ In general, the elongation of the JT-distorted octahedra diminishes under compression (referred to as Jahn–Teller suppression), which sometimes appears to be the driving force for phase transitions,^4^ but this is not general.^5^ Another interesting point of study—of particular relevance for perovskite-like systems—is whether JT suppression or displacive distortions, such as octahedral tilting, dominate the compression of the compound.^3,6–8^ These studies enhance the understanding of the degrees of freedom present in a material and ultimately improve the ability to design and optimise properties for various applications.

JT distortions are also important in molecular complexes—they are often exploited in molecular magnetism^9^ and can facilitate the adoption of polar space groups.^10^ An intriguing pressure effect is so-called Jahn–Teller switching, where the long axis of a (pseudo)-JT octahedron changes from one pair of ligands to another under pressure. A key example is CuF_2_(H_2_O)_2_(pyrazine), where the JT switching changes the dimensionality of the magnetic coupling from 2D to 1D.^11^ Similar behaviour occurs in a few other coordination polymers,^12–14^ and in the archetypal Tutton's salt, (ND_4_)_2_[Cu(D_2_O)_6_][SO_4_].^15^ While this phenomenon may have other causes than the pure electronic effects of a JT effect,^16^ it is intriguing and has functional implications. Even less dramatic changes, such as the anisotropic compressibilities typical of JT-distorted octahedra, have been linked to applications within spintronics^17^ and piezochromism.^13^

Deconvoluting the roles played by different degrees of freedom—such as a JT distortion or octahedral tilting—in the presence of others is challenging. This is perhaps especially prominent in molecular frameworks, which have greater structural flexibility and more degrees of freedom than *e.g.* ceramics.^18^ Hence, it is difficult to make general statements about how a JT distortion impacts the pressure behaviour of a molecular framework. Consequently, the design principles for JT switching and the role of JT suppression as a trigger of phase transitions—to just use two examples—are poorly understood. There would be great benefit to further high-pressure studies focused on understanding the pressure response of JT distortions and the extent to which this can be generalised.

Prussian blue analogues (PBAs)^19,20^ are a useful model system for systematic studies with varying degrees of freedom. Their generic formula can be expressed as A_*x*_M[M′(CN)_6_]_1−*y*_□_*y*_·*n*H_2_O, where A is an alkali metal ion, M/M′ are normally transition metals, and □ refers to a M′(CN)_6_ vacancy (defect). The coefficients *x* (0 ≤ *x* ≤ 2) and *y* (0 ≤ *y* ≤ 1/3) stipulate the stoichiometry, *i.e.* the A-site occupancy and amount of M′-site vacancies. On account of the tunability enabled by varying *x* and *y*, PBAs are truly versatile materials with properties ranging from those commonly associated with open frameworks—*e.g.* catalysis^21,22^ and gas storage ability^23,24^—to those typical for ceramics, including magnetism^25,26^ and ion transport.^27,28^ The latter property renders PBAs of interest as next-generation electrode materials. PBAs bridge the gap between perovskites and coordination polymers/metal–organic frameworks (MOFs); so their behaviour may have a bearing on both of these topical families. The tunability allows the study of JT distortions as other degrees of freedom are varied, *e.g.* porosity, hydration, and the nature of interstitial ions. This will advance the understanding of JT distortions and their interplay with other aspects of the crystal structure.

The archetypal JT-distorted ion is Cu^II^, and Cu^II^-based PBAs are interesting in their own right. As the Cu^II^–NC bond is particularly strong, these PBAs often exhibit extreme properties, including stronger magnetic coupling and lower hydration levels than related systems.^29^ Cooperative JT distortions, so-called orbital order, also lower the symmetry in *e.g.* MPt(CN)_6_ if M = Cu^II^, which affects the mechanical properties.^30,31^ Moreover, the JT distortions can influence other degrees of freedom: single crystals of Cu[M′(CN)_6_]_0.67_ show an extreme local arrangement of the M′(CN)_6_ vacancies relative to non-JT-distorted analogues.^32^ Likewise, the Rb^I^ ion order in RbMM′(CN)_6_ is dictated by whether M = Cu^II^ or the JT-inactive Mn^II^.^33–35^ As highlighted, Cu-PBAs show a large structural diversity, and thus form an especially intriguing subset of PBAs, worthy of further study.

**Fig. 1:**
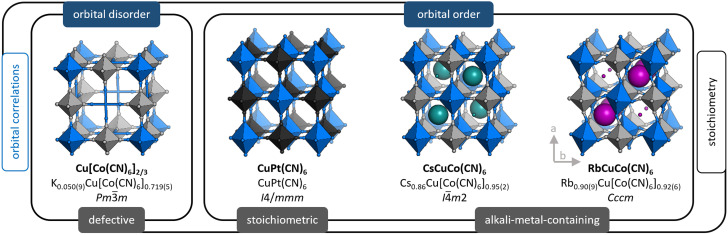
The crystal structures of the PBAs relevant to this study, with the ideal formula (bold), the composition as found by ICP, and the space group. The structures are categorised based on whether the Jahn–Teller distortions are disordered or ordered (blue labels, top) and their stoichiometry (grey label, bottom). The size of the alkali metal ions is proportional to their occupancy. Note that the M′ vacancy in Cu[Co]_0.67_ is disordered in reality, but an ordered arrangement is shown for clarity.

We use X-ray and neutron diffraction (XRD/ND) to investigate the high-pressure behaviour of a series of JT-distorted PBAs [Fig. 1], namely the defective Cu[Co(CN)_6_]_0.67_, the stoichiometric CuPt(CN)_6_ and the alkali-metal-containing ACuCo(CN)_6_ (A = Cs^I^, Rb^I^). While all compounds feature JT distortions, this manifests differently depending on the other degrees of freedom. Cu[Co(CN)_6_]_0.67_ shows uncorrelated JT distortions—orbital disorder—and the average symmetry is cubic. In CuPt(CN)_6_ and ACuCo(CN)_6_, the long axes of the JT distortions align and reduce the symmetry to at least tetragonal. Hence, the structural complexity gradually increases across the series: CuPt(CN)_6_ is a simple, JT-distorted ReO_3_ analogue,^30^ CsCuCo(CN)_6_ contains Cs^I^ ions at half the interstitial A-sites—which lifts the inversion symmetry^36^—and RbCuCo(CN)_6_ features both A-site cations and octahedral tilts.^33,34^ As Cu[Co(CN)_6_]_0.67_ and CuPt(CN)_6_ are porous, they were studied both in the as-synthesised (hydrated) and dehydrated forms. Our study thus explores the pressure response of JT distortions—both cooperative and disordered—in the presence of other degrees of freedom: vacancies, A-site cations, octahedral tilts, and hydration.

The manuscript commences by discussing the results of the samples in order of increasing complexity. Cu[Co(CN)_6_]_0.67_·*n*H_2_O remains crystalline up to at least 3 GPa, whereas the dehydrated Cu[Co(CN)_6_]_0.67_ amorphises below 1 GPa. The stoichiometric CuPt(CN)_6_·*n*H_2_O and CuPt(CN)_6_ both undergo crystalline–crystalline phase transitions and ACuCo(CN)_6_ remain in their ambient phases up to nearly 3 GPa, where RbCuCo(CN)_6_ transitions to a monoclinic phase. The discussion compares the phase transitions and compressibilities of the studied compounds, and comparison with the behaviour of the corresponding Mn-based PBAs^37^ enables the effect of the JT distortions to be identified. Previous studies indicate that the pressure response of PBAs is not strongly dependent on the radius of the M metal;^37^ hence, the differences between Mn- and Cu-based PBAs can be directly attributed to the JT distortions.

## 2 Results

The compositions of the samples as found by inductively-coupled plasma (ICP) [see ESI† for experimental details] are shown in Fig. 1 along with the space group at ambient conditions. For brevity, we hereafter refer to these as *e.g.* Cu[Co]_0.67_ and CuPt, where [Co] = [Co(CN)_6_] and Pt = Pt(CN)_6_.

### 2.1 Cu[Co(CN)_6_]_0.67_

Both as-synthesised Cu[Co]_0.67_·*n*H_2_O and dehydrated Cu[Co]_0.67_ adopt cubic symmetry with orbital disorder, as the vacancies disrupt the correlations between the JT distortions. Defective PBAs with (seemingly) random vacancy arrangements crystallise with *Fm*3̄*m* symmetry, on average, but Cu[Co]_0.67_ shows superstructure reflections indicative of *Pm*3̄*m*. Primitive cubic symmetry is a signature of a non-random vacancy distribution and originally observed in Prussian blue itself,^19^ but also frequently seen in defective Cu-PBAs.^38,39^ The superstructure reflections are less evident in Cu[Co]_0.67_·*n*H_2_O, as water molecules occupy the vacancies and reduce the contrast in scattering power between occupied and vacant sites. Yet, the additional intensity around 2*θ* = 5° for Cu[Co]_0.67_·*n*H_2_O [Fig. S9†] could be the result of diffuse scattering from partial vacancy order. Although, scattering from the pressure-transmitting medium (PTM) typically appears in this region, which also could contribute to the observed feature.

**Fig. 2:**
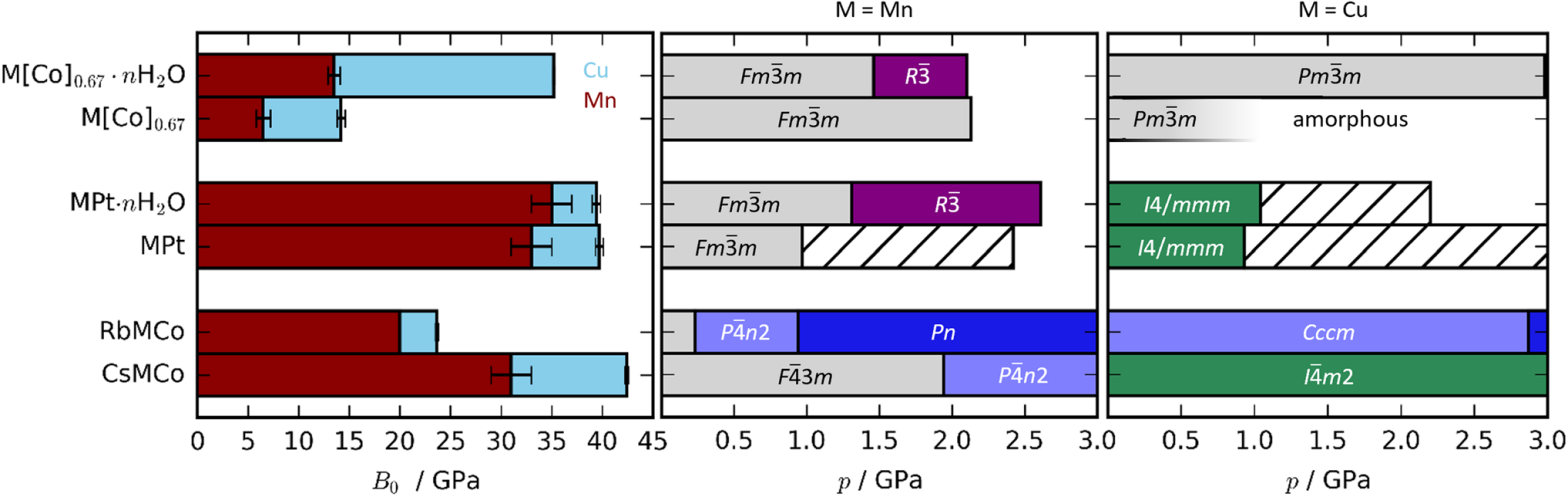
(Left) Bulk moduli of various PBAs where the values for the Cu-based and Mn-based PBAs are indicated in blue and red, respectively. Note that the bulk modulus of RbMnCo(CN)_6_ is calculated based on solely two data points. (Right) The phase diagrams of Cu- and Mn-based PBAs where hashed bars denote unsolved phases. The data for the Mn-PBAs are taken from ref. 31, 37 and 50.

The presence/absence of interstitial guest water has consequences for the cell volume and compressibility. Cu[Co]_0.67_ has a smaller lattice parameter (*a* ∼ 9.96 Å) at ambient pressure relative to Cu[Co]_0.67_·*n*H_2_O (*a* ∼ 10.5 Å), evident of a contraction of the unit cell upon dehydration [Fig. S19†]. Such breathing effects are common in porous and flexible materials and the magnitude observed here is comparatively small.^40,41^ The bulk moduli—given by 
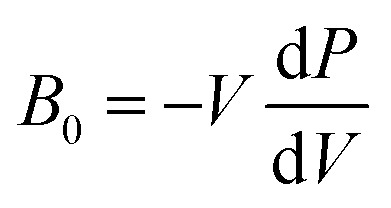
 and hence inversely related to the volume compressibility—were calculated by second-order Birch–Murnaghan^42–44^ fits as 14.2(4) GPa and 35.2(11) GPa for Cu[Co]_0.67_ and Cu[Co]_0.67_·*n*H_2_O respectively. Water thus increases the stiffness of the framework by a substantial amount [Table 1, Fig. 2], similar to what occurs in MOFs.^45,46^ Altogether, the hydration effects in porous PBAs agree with previous studies.^31,47^

**Table 1:** The Prussian blue analogues investigated here, including the composition, space group, phase transition pressure (*p*_T_), the bulk moduli (*B*_0_) from second-order Birch-Murnaghan fits,^42,43^ and the uniaxial compressibilities.^48^ The transition pressure is calculated as the average between the pressure before and after the transition. Roman numerals denote different phases

Sample	Space group	*p*_T_/GPa	*B*_0_/GPa	*K*_*a*_/TPa^−1^	*K*_*b*_/TPa^−1^	*K*_*c*_/TPa^−1^	Radiation
Cu[Co]_0.67_·*n*H_2_O	*Pm*3̄*m*	35.2(11)	8.38(10)	—	—	X-ray	
Cu[Co]_0.67_	*Pm*3̄*m*	∼0.7	14.2(4)	22(2)	—	—	X-ray
CuPt·*n*H_2_O	*I*4/*mmm*	1.04(2)	39.4(4)	4.7(2)	—	14.7(5)	X-ray
CuPt	*I*4/*mmm*	0.92(5)	39.7(4)	4.82(8)	—	14.0(2)	X-ray
CuPt-III	*Pm*3̄*m*		2.6(6)	36(8)	—	—	X-ray
CsCuCo	*I*4̄*m*2	42.4(2)	4.12(5)	—	12.75(10)	X-ray	
CsCuCo	*I*4̄*m*2	38.5(8)	4.8(4)	—	13(1)	Neutron	
RbCuCo	*Cccm*	2.9(2)	23.66(15)	23.1(2)	8.77(4)	3.94(3)	X-ray
RbCuCo	*Cccm*	—	25.8(5)	17.0(9)	9.2(2)	3.4(3)	Neutron
RbCuCo-II	*P*2/*m*	—	—		—	X-ray	

The phase behaviour upon compression also varies between Cu[Co]_0.67_ and Cu[Co]_0.67_·*n*H_2_O. The latter compresses uniformly without significant changes to the XRD patterns, whereas the crystallinity of Cu[Co]_0.67_ rapidly decreases and the sample amorphises below 1 GPa [Fig. S1 and S2†]. The crystalline state could not be recovered upon decompression, thereby confirming irreversible pressure-induced amorphisation (PIA) of Cu[Co]_0.67_. This is in line with the recent observation that a glassy state of the related Cu[Fe(CN)_6_]_0.67_ can be obtained *via* ball milling.^49^ The resulting PBA glass recrystallises when exposed to humid air,^49^ which is interesting given that Cu[Co]_0.67_·*n*H_2_O remains crystalline upon compression. Clearly the presence of water can both prevent and reverse mechanically induced amorphisation.

### 2.2 CuPt(CN)_6_

CuPt and CuPt·*n*H_2_O crystallise in space group *I*4/*mmm*, which can be derived from the cubic parent PBA structure by elongating along *c* due to a cooperative JT distortion. The *c* lattice parameter is ∼0.03 Å shorter in CuPt than CuPt·*n*H_2_O, indicating a small tetragonal compression upon dehydration—a considerably smaller contraction than in the defective Cu[Co]_0.67_. The samples compress uniformly up to *ca.* 1 GPa with nearly identical bulk moduli of 39.7(4) and 39.4(4) GPa for CuPt and CuPt·*n*H_2_O, respectively. The uniaxial compressibilities (*K*_*i*_) are highly anisotropic [Table 1 and Fig. S20†], with *K*_*c*_ ∼ 14–15 TPa^−1^ and *K*_a_ ∼ 5 TPa^−1^. A soft *c*-axis is typical for compounds with a JT distortion along *c*, as the long axis is more compressible than the perpendicular equatorial plane.^17,51,52^ Overall, the compressibilities for CuPt·*n*H_2_O and CuPt are identical within error to previously obtained values,^31^ and hydration does not significantly influence the mechanical properties of CuPt.

Around 1 GPa, new reflections appear in the XRD patterns of both CuPt and CuPt·*n*H_2_O [Fig. S3 and S4†], pointing towards first-order phase transitions. The high-pressure and ambient phases coexist up to ∼1.3–1.4 GPa, where the transition is complete. Although the XRD patterns of the high-pressure phases are very similar, the pattern of CuPt contains extra reflections not observed for CuPt·*n*H_2_O, suggesting that the phase transitions could be dictated by the water content. Similar effects, where water changes the phase transitions, are known for the analogous MnPt^37^ and for the transitions induced by sodiation—*i.e.* the application of negative pressure—of *e.g.* Na_*x*_MnFe(CN)_6_.^53^ Despite our best efforts, attempts at solving the high-pressure phases were unsuccessful. Unlike previous reports,^31^ the phase transitions and compression appear independent of the X-ray exposure, although this may result from the deliberate attenuation of the X-ray beam during the experiment.

CuPt·*n*H_2_O remains in the (unsolved) high-pressure phase until 2 GPa and reverses to the ambient phase upon decompression, whereas CuPt behaves less straightforwardly. As the pressure increases after the transition at 1 GPa and most diffraction peaks broaden, certain reflections increase in intensity —most notably at 2*θ* ∼ 5.2°. At 3 GPa, the intensity of the reflections from this third phase dominates over the other phase. Decompression recovers the tetragonal phase, but the reflections corresponding to the third phase remain. Visual inspection of the sample after the experiment also revealed two phases: one black and one white [Fig. S13†]. Collectively, this indicates that CuPt undergoes a partial structural collapse followed by separation into two phases. Possible explanations for the phase separation could include reaction of the sample with the sample holder, the presence of an impurity that becomes more noticeable as the pressure increases, or a strain-induced phase transition resulting from non-hydrostatic conditions.

A space group and bulk modulus were found for the phase-separated form of CuPt-III. It can be indexed in *Pm*3̄*m* with *a* ∼ 5.8 Å at 3.32 GPa, which is a surprising ascent in symmetry from the ambient tetragonal crystal system to a cubic phase. The unit cell parameter is similar to the Cu–Pt distance in cubic PBAs and larger than what would be expected of a simple metal or CuPt alloy.^54^ The bulk modulus is 2.6(6) GPa in the range 1.4–2.1 GPa, which is considerably softer than the ambient phase. The lattice parameters vary slightly between different sample positions in this pressure range, which might relate to different degrees of structural collapse throughout the sample. Above 2.2 GPa, the lattice parameters are unchanged even as the pressure increases, likely due to non-hydrostatic conditions.^55^

### 2.3 ACuCo(CN)_6_ (A = Cs, Rb)

Despite the related compositions, the ambient crystal structures of RbCuCo and CsCuCo are distinctly different [Fig. 1],^34^ as first noticed for the analogous hexacyanoferrates.^33^ The tetragonal, non-centrosymmetric structure of CsCuCo can be derived from the *I*4/*mmm* symmetry of CuPt, by adding Cs^I^ cations to the interstitial sites in an alternating fashion. This Cs^I^ arrangement lowers the symmetry to the non-centrosymmetric space group *I*4̄*m*2. In contrast, RbCuCo features a columnar order of the Rb^I^ ions along *c* accompanied by in-phase octahedral tilting (*a*^0^*a*^0^*c*^+^ in Glazer notation^56^) polarised along the same axis. This leads to the orthorhombic space group *Cccm*, with the cooperative Jahn–Teller distortion along *a*.^34^

The pressure response of CsCuCo partially mirrors that of the hexacyanoplatinates—while the lattice parameters are smaller in absolute terms, the compressibility found by XRD is similarly anisotropic with *K*_*a*_ = 4.12(5) TPa^−1^ and *K*_*c*_ = 12.75(10) TPa^−1^ [Table 1]. The bulk modulus is 42.4(2) GPa, which is ∼3 GPa larger than for the hexacyanoplatinates on account of the slightly stiffer *c*-axis. ND data give a slightly lower value for the *c* axis and a larger volume compressibility (lower bulk modulus) [Table 1]. Given difficulties in accurately modelling the peakshape and the many overlapping peaks in the ND data, the values obtained from the XRD data are more reliable. No obvious phase transitions were observed for CsCuCo up to ∼4.8 GPa [Fig. S5 and S6†].

RbCuCo also remains in its ambient space group (*Cccm*) up to ∼3 GPa with anisotropic compression [Fig. S7 and S8†]. Based on the results of the XRD refinements, the compressibilities increase in the order *K*_*a*_ = 23.1(2) TPa^−1^ > *K*_*b*_ = 8.77(4) TPa^−1^ > *K*_*c*_ = 3.94(3) TPa^−1^ [Table 1 and Fig. S21†], which can be rationalised based on the structural degrees of freedom. The main mechanisms for volume reduction are increased octahedral tilting and compression of the JT distortion—as seen in the tetragonal samples. Both of these will shorten *a*, thereby leading to a large compressibility along this axis, whereas *b* is only compressed by enhanced tilting, and neither compression mechanism affects *c*. As a result, *K*_*c*_ is very low (∼4 TPa^−1^), similarly to the *a*-axis of the tetragonal PBAs. This order of flexibility of the axes mirrors the thermal expansion, where *a* features large positive expansion and *c* shows a small negative thermal expansion.^34^ The trend agrees with a recent computational study,^57^ although the absolute values differ by a factor of ∼4. The bulk modulus of RbCuCo is ∼24 GPa, which is substantially lower than for CsCuCo, and highlights the importance of octahedral tilting as a mechanism for compression.

**Fig. 3:**
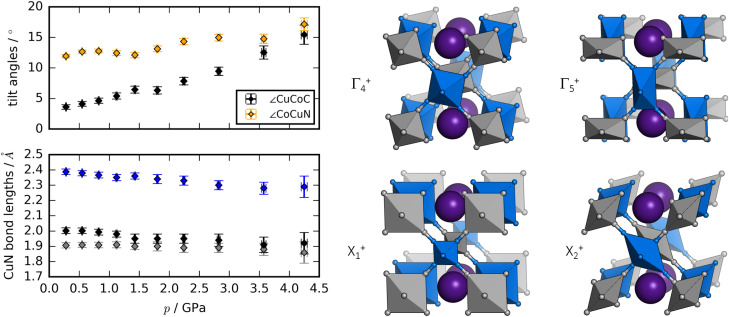
The left panel shows the pressure evolution of the tilt angles of RbCuCo, with ∠CuCoC in black and ∠CoCuN in orange, and the CuN bond lengths of RbCuCo as a function of pressure. The right panel visualises the distortion modes (with exaggerated amplitudes) potentially driving the phase transition in RbCuCo.

Rietveld refinement of ND data^58,59^ allows a deeper insight into the structural changes during compression [Fig. 3]. The dominant distortion during compression is increased magnitude of the octahedral tilting (*a*^0^*a*^0^*b*^+^). In particular, the tilt angle of the CoC_6_ moiety—defined as ∠CuCoC—is sensitive to pressure and increases from ∼3° to ∼15° from 0 to 4 GPa. The CuN_6_ group already has a sizaeble tilt magnitude at ambient conditions (∼12°), which remains largely constant with pressure, although it is worth being mindful of the considerable uncertainties associated with bond angles. Nevertheless, the observations regarding the relative tilt angles agree with the idea that the M′–C–N bond has a larger preference for linear geometry relative to the M–N–C moiety, due to the strong π-back bonding of the CN ligand.^61^

As expected, the JT distortion of RbCuCo decreases with pressure. The orthorhombic symmetry leads to three pairs of Cu–N bond lengths, which at the lowest pressure point (0.3 GPa) refine to 1.91(2), 2.00(2), and 2.39(2) Å, respectively. Upon compression, they decrease with compressibilities related to the bond length—the longest bond is unsurprisingly the most compressible [Fig. 3]. This is in line with the discussion for the tetragonal CuPt samples. However, in terms of absolute magnitude, the tilt distortions are greater than the JT distortion and dominate the compression mechanism, leading to a softening relative to the CuPt family.

At ∼3 GPa, several reflections in the XRD pattern of RbCuCo split, most notably the intense (110) peak [∼3° in Fig. S7 and S18†]. No splitting was evident in the neutron diffraction data, yet the pressure trend of the tilt angles and bond lengths diverge around 3 GPa and there is a small discontinuity in the pressure evolution of the *a* axis [Fig. S21†]. This could result from a phase transition at this pressure, but the lower resolution and substantial peak broadening make compelling conclusions difficult. The discrepancy may also result from the use of different pressure-transmitting media (Daphne oil *vs.* methanol : ethanol). Alternatively, the transition is simply not visible in the ND data due to peak broadening.

The peak splitting observed in the XRD data of RbCuCo is consistent with the subgroup *P*2/*m* with lattice parameters (*a*_m_, *b*_m_, *c*_m_) related to those of the parent *Cccm* structure (*a*_0_, *b*_0_, *c*_0_) as 
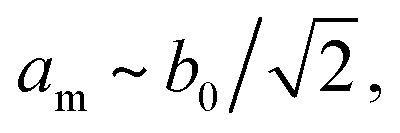
*b*_m_ ∼ *c*_0_, and 
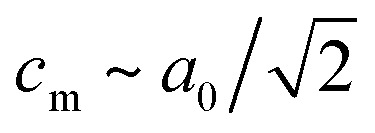
 [Fig. S18†]. Using the software ISODISTORT,^60^ one (and only one) subgroup of *Cccm* with the correct space group *P*2/*m* and basis can be identified. The possible primary order parameters for this transition are Γ^+^_4_ (octahedral tilt), Γ^+^_5_ (octahedral deformation), X^+^_1_ (alternating octahedral strain), and X^+^_2_ (alternating octahedral elongation) [Fig. 3]. The irreducible representations are given relative to the *Fm*3̄*m* parent structure. Refinement of the low-symmetry phase was attempted, but reliable positional parameters could not be extracted, due to the low sensitivity to the cyanide positions (for the XRD data) or the low resolution (for the neutron data).

While the dominant distortion driving the *Cccm*–*P*2/*m* transition cannot be identified with certainty, educated speculation is possible. For example, the distortion denoted Γ^+^_5_ corresponds to an octahedral deformation or a translation of the Rb^I^ ions towards each other, which are likely associated with a high energy cost. Likewise, the X^+^_1_ mode creates two very unequal environments for chemically equivalent groups and leads to the same pairwise translation of Rb^I^ ions. However, Γ^+^_4_ describes octahedral tilting—here polarised along *b*—which is well documented in PBAs,^60^ and could be a plausible option. Γ^+^_5_ is also interesting: this mode corresponds to an alternating JT distortion and if this drives the transition, RbCuCo joins the category of samples with pressure-induced JT orientations. All distortions preserve the linear arrangement of Cu–N–C–Co along ***b*** (due to the presence of a 2-fold axis along ***c***) and so tilts polarised along ***a*** or ***b*** are not present.

## 3 Discussion

**Fig. 4:**
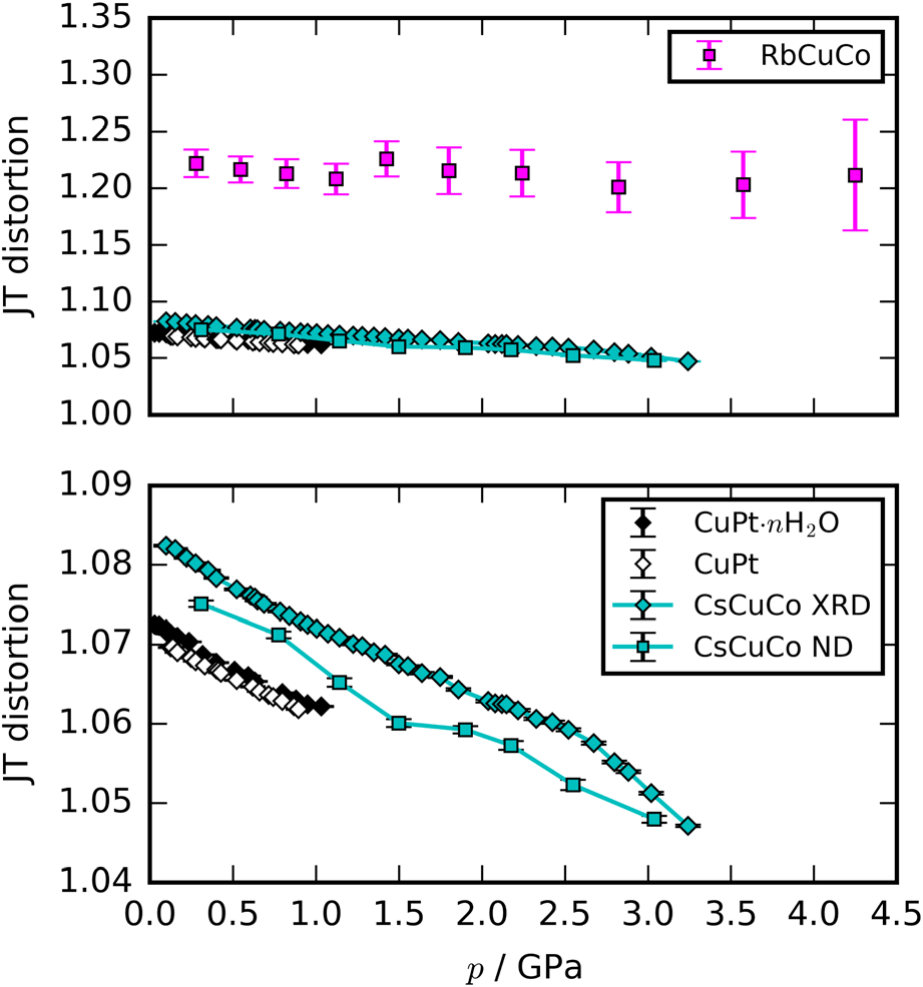
The magnitude of the JT distortion as a function of pressure for the orbitally ordered samples. The upper panel includes all the studied PBAs, whereas the lower shows a close-up view. The JT elongation for RbCuCo is calculated as ratio of the long axis and the average of the two shorter axes.

Most high-pressure studies on JT-distorted compounds report that the magnitude of the JT distortion decreases with pressure.^3,8,62,63^ For the tetragonal PBAs studied here, the magnitude of the JT distortion is equal to 
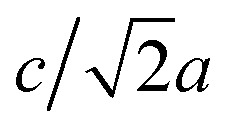
—assuming negligible changes of the CN bond during compression—and, for RbCuCo, neutron diffraction allows the JT elongation to be calculated from the Cu–N bond lengths [Fig. 4]. The absolute value of the JT elongation at ambient pressure is dictated by the composition of the PBA, with the largest value for RbCuCo (∼1.23), and a slightly larger elongation in CsCuCo (∼1.08) than in CuPt (∼1.07). The difference between RbCuCo and the other PBAs may be due to the tilting, but the difference in methods (direct refinement of bond lengths *vs.* refinements of lattice parameters) could also play a role. Dehydration decreases the JT magnitude slightly in CuPt relative to CuPt·*n*H_2_O. By way of comparison, the JT elongation in the Cu-MOFs HKUST-1 and Cu-pyr are ∼1.1 and ∼1.17 at ambient conditions.^13,64^ The variation in Cu^II^–ligand bond lengths showcases the flexibility of the JT distortion and its ability to adapt to different crystal structures and local environments.

### 3.1 Compressibility

The compression of the JT distortions proceeds at largely the same rate in most of the PBAs, apart from RbCuCo [Fig. 4 and Table 1]. The compressibilities of CuPt and CuPt·*n*H_2_O are identical within error, indicating that there is no significant effect of water on the mechanical properties of these systems. CsCuCo features a slightly stiffer *c*-axis, which can be attributed to the bulky Cs^I^ ions, but this effect is minor. The softer JT distortion found by neutron diffraction is less reliable, but included for completeness. In RbCuCo, the compression can proceed both *via* the JT distortion and by increasing the magnitude of the tilts. Octahedral tilting dominates the compression and this sample displays a less compliant JT distortion than the untilted PBAs. To summarise, the volume reduction mechanisms of JT-ordered tetragonal PBAs are governed by compression of the JT distortions, with other degrees of freedom playing a secondary role—except for octahedral tilting.

The trend in compressibility as a function of composition/hydration is qualitatively similar for both Cu-PBAs and Mn-PBAs,^37^ but some deviations are obvious [Fig. 2]. First, the bulk moduli of Cu-PBAs are invariably 5–20 GPa greater than the values of the Mn analogues. Although JT distortions lead to a softening along one direction, they also decrease the compressibility along the remaining axes, giving a net stiffening effect. The inverse scenario or no change is seen in perovskites,^62,63^ and in some coordination polymers, the presence of Cu^II^ appears to give a small softening.^65,66^ Second, the bulk modulus of Cu[Co]_0.67_·*n*H_2_O is three times as large as that of Mn[Co]_0.67_·*n*H_2_O—almost on par with the values of the stoichiometric hexacyanoplatinates. In other words, the softening effect of vacancies—clearly seen by comparing Mn[Co]_0.67_ and MnPt—is completely cancelled out by introducing disordered JT distortions. This points towards a coupling between disordered JT distortions and the volume reduction mechanisms.

Turning to Cu[Co]_0.67_ and Cu[Co]_0.67_·*n*H_2_O, the compressibility is influenced by both the hydration and the [Co(CN)_6_] vacancies. Cu[Co]_0.67_ is noticeably softer than both CuPt and RbCuCo [Fig. 2], affirming that defects are the degree of freedom with the largest impact on the mechanical properties—even greater than the octahedral tilting.^37^ Moreover, while interstitial water molecules have a negligible effect on the compression of CuPt and CuPt·*n*H_2_O, Cu[Co]_0.67_ is more than twice as compressible as its hydrated analogue Cu[Co]_0.67_·*n*H_2_O. On one hand, this is unsurprising, as guest inclusion in porous frameworks such as MOFs normally increases the resistance to compression.^45,46^ Yet, on the other hand, the magnitude of the change is extremely large, especially in comparison to the Mn analogues. This shows that, while Cu-based PBAs have a comparatively low affinity to guest water,^29^ hydration/dehydration is still an important factor to consider.

It is worth speculating on possible reasons behind the anomalously high stiffness of Cu[Co]_0.67_·*n*H_2_O in comparison to Mn[Co]_0.67_·*n*H_2_O and Cu[Co]_0.67_. As the compositions of the Mn- and Cu-PBAs are very similar,^37^ variations in vacancy content can be ruled out as the cause. Likewise, Cu-PBAs are known to feature lower hydration degrees than other PBAs^29^—which should increase the compressibility—and so deviating water content also does not rationalise the observed differences. It follows that the stiffening of Cu[Co]_0.67_·*n*H_2_O is directly related to the JT distortion and its disordered state, and is somehow enhanced by the presence of water molecules. Given the three-fold disorder present in this system—vacancies, JT distortions, and water—understanding the unusually high resistance to compression will be challenging, and requires further study of the local structure.

A-site cations exert a minor influence on the mechanical properties of the studied PBAs. CsCuCo is the stiffest compound, which is in line with this being the densest PBA with the least void space [Fig. 2]. Replacing Cs^I^ with Rb^I^ leads to a substantial softening by a factor of ∼2. The large dependence on the alkali metal is a consequence of the different symmetries: the octahedral tilts in RbCuCo provide an additional mechanism for compression not present in CsCuCo. CuPt and CuPt·*n*H_2_O are only marginally softer than the Cs-containing analogue, indicating that the Cs^I^ ion has a minor influence on the mechanical properties, as discussed above. Therefore, octahedral tilts are clearly the most influential design element to enhance the stiffness of a non-defective PBA, whereas the intrinsic effects of hydration and alkali metal cations are considerably lower.

### 3.2 Phase transition behaviour

For defective PBAs, the phase transitions under pressure are strongly influenced by the presence or absence of JT distortions. Mn[Co]_0.67_·*n*H_2_O shows a *Fm*3̄*m*–*R*3̄ transition,^37^ not observed in the Cu analogue; yet while Mn[Co]_0.67_ remains cubic up to at least 2 GPa,^37^ Cu[Co]_0.67_ amorphises below 1 GPa. In other words, the JT distortion prevents crystalline-to-crystalline phase transitions in the presence of water, yet drastically reduces the critical pressure for amorphisation in dehydrated PBAs. Cu[Co]_0.67_ amorphises earlier than its Mn counterpart, yet is less compliant. This indicates that the disordered JT distortion—for reasons yet unclear—prevents the access to a useful compression mechanism, and hence amorphisation is the favoured route to volume reduction. In summary, JT distortions have a substantial impact on the phase transitions of the defective (and JT-disordered) PBAs.

Amorphous Cu[Co]_0.67_ obtained at pressures below 1 GPa can be recovered to ambient pressure. Amorphous and glassy coordination polymers have been attracting growing interest during the last decades, as the disordered nature can improve the ion conductivity and alter the mechanical and optical properties.^67–69^ However, the first solid amorphous PBA—mechanochemically amorphised Cu[Fe(CN)_6_]_0.67_—was only recently reported.^49^ Our findings (unsurprisingly) indicate that amorphisation under pressure is general for defective Cu-based PBAs, regardless of the nature of the M′-site ion. As the amorphisation of Cu[Fe(CN)_6_]_0.67_ changed its electronic properties and sorption behaviour,^49^ further study of the structure and properties of amorphous PBAs will be rewarding.

Pressure-induced amorphisation (PIA)—as displayed by Cu[Co]_0.67_—has potential functional implications for coordination polymers. For example, PIA can be used to trap hazardous gases,^70^ and may serve as a shock-absorbing mechanism.^71^ Many molecular frameworks typically display a relatively low critical pressure for amorphisation (*p*_a_), which can be tuned further by the guest species.^71–73^ Our results mirror this, as Cu[Co]_0.67_·*n*H_2_O remains crystalline to higher pressures than Cu[Co]_0.67_ (*p*_a_ < 1 GPa). Thinking towards design principles, PIA is believed to be linked to the presence of negative thermal expansion (NTE)^74,75^ and Cu[Co]_0.67_ indeed belongs to the class of NTE materials, with a coefficient of thermal expansion of −20(2) MK^−1^.^76^ Furthermore, the vacancies in Cu[Co]_0.67_ appear critical for the PIA, as the stoichiometric (vacancy-free) FeCo(CN)_6_ has a *p*_a_ of ∼10 GPa.^77^ Additional studies to higher pressures are needed to fully uncover the compositional effect on PIA in PBAs.

Alkali metals reduce the need to accommodate the effects of pressure *via* a phase transition in JT-distorted PBAs, or at least increase the critical pressure [Fig. 2]. Mn-based PBAs with alkali metals undergo at least one phase transition below 2 GPa—RbMnCo at pressures as low as 0.2 GPa ^37^—whereas RbCuCo and CsCuCo remain in their ambient phases until at least 3 GPa. This is likely due to the fact that JT distortions provide an additional compression mechanism, so that volume reduction can proceed to higher pressures without a change in global symmetry. However, the presence of octahedral tilts in RbCuCo means that the lower predisposition to phase transitions cannot be solely attributed to the JT distortions. Any effects of the JT distortion are less evident in the stoichiometric MPt systems, as all four PBAs undergo transitions around 1 GPa regardless of the identity of M. For context, the control of phase transitions is highly relevant within *e.g.* the electrochemical community,^78,79^ where PBAs are intensely studied.^27^ Altogether, the combination of alkali cations and JT distortions is a recipe for phase transition prevention.

### 3.3 Outlook

It is instructive to compare the pressure response of the PBAs with that of related compounds. Double perovskites of formula A_2_CuB′O_6_ (B′ is a heavy transition metal) feature the same framework topology and orbital order as the present PBAs and are therefore good comparison systems. However, the tilts differ: our PBAs are either untilted or tilted in phase along one axis (*a*^0^*a*^0^*b*^+^ in Glazer notation^56^) perpendicular to the JT distortion, whereas A_2_CuB′O_6_ tilt out-of-phase (*a*^0^*a*^0^*b*^−^) along the same axis as the JT elongation.^52,62^ Unlike the PBAs, the compressibilities of the perovskites do no display any obvious dependence on whether JT distortions are present or not, indicating that other factors dominate.^52,62^ Furthermore, JT suppression is the key compression mechanism in the perovskites,^52,62^ whereas it is secondary to the octahedral tilting in RbCuCo—the only PBA with both JT distortions and tilts. These differences may arise from differences in tilt systems or in the degrees of covalency in the bonds. It follows that the topology does not necessarily dictate the pressure response—at least not in this particular case.

Many studies have tried to relate the extent of the JT suppression to the presence of phase transitions. JT distortions typically break the global symmetry, so complete suppression—giving a CuL_6_ octahedron with *O*_h_ symmetry—would drive a transition. This is also observed in *e.g.* Rb_2_CuCl_2_Br_2_, where the pressure-induced transition is concomitant with complete JT suppression.^4^ However, it is not a general phenomenon: cuproscheelite undergoes a transition when the Cu–O bonds are still of unequal length,^5^ by way of example. Returning to our PBAs, CuPt and CuPt·*n*H_2_O retained some JT elongation (∼1.06) at the transition point, whereas CsCuCo reached a greater extent of compression (<1.05) without a phase transition—although the XRD data are in the non-hydrostatic regime, which reduces the reliability. The presence of A-site cations hence stabilises the structure against transitions, without affecting the JT compression. Extrapolating the JT compression of CsCuCo indicates that a phase transition should occur before ∼9 GPa, as this is where the JT distortion would be completely suppressed. Consequently, there does not appear to be a universal threshold value of the JT elongation, beyond which a phase transition is required.

## 4 Conclusion

To conclude, the pressure response of Cu^II^-based Prussian blue analogues is strongly impacted by the Jahn–Teller distortion. JT-distorted PBAs universally show higher bulk moduli than the JT-inactive counterparts, which is particularly pronounced in the orbitally disordered Cu[Co]_0.67_·*n*H_2_O. The phase transition behaviour also changes as JT distortions are introduced. Generally, Cu-based PBAs with A-site cations appear less susceptible to pressure-induced crystalline-to-crystalline phase transitions relative to the JT-inactive counterparts, as compression of the JT distortion itself acts as a volume-minimising mechanism and reduces the need for phase transitions. Nevertheless, the presence of Cu^II^ increases the propensity for amorphisation under pressure in M[Co(CN)_6_]_0.67_. This demonstrates the important role of JT distortions in the development of structure–property relationships.

Our results highlight the need for further studies into the local structures of PBAs, for example using vibrational spectroscopy or total scattering. This will elucidate the role of the active distortions at play during compression, which is particularly important for the orbitally disordered Cu[Co]_0.67_, where the largest effect of the JT distortions were seen. It is worth emphasising that orbital disorder is rare in coordination polymers, yet is implicated in *e.g.* the giant magnetoresistance in perovskite manganites.^2^ Hence, any fundamental understanding of the orbital correlations in Cu[Co]_0.67_ may also have a bearing on this field. While local structure studies are challenging, important insights into local order in PBAs have been obtained using analysis of diffuse scattering.^32^ In addition, the development of high-pressure total scattering measurements is encouraging in this context,^80–82^ as it will allow the JT suppression to be studied in more detail—even for disordered samples.

Understanding the pressure response is important for several fields of future applications for PBAs. They are important materials for the development of next-generation batteries,^27^ and so the behaviour under external hydrostatic pressure could help understand the effect of the internal chemical pressure that occurs during battery cycling. Moreover, the information about the bulk moduli complements the relatively well-explored field of (negative) thermal expansion in PBAs.^30,76,83,84^ Lastly, knowledge about the structural integrity under pressure is critical for the exploitation of pressure-induced switchability—including charge transfer^85,86^ or spin crossover^87–89^—as well as for the design of amorphous states.^49^ The fundamental findings of this study are therefore also interesting from an applied perspective.

As a final point, it is worth looking towards the broader field of JT distortions under pressure. Generalising our results to other compounds is nontrivial, and the interplay between JT distortions and the pressure response appears to be highly material dependent. Yet, the strong effects discussed herein can still serve as a benchmark for future studies. Additional research may also nuance the understanding of various aspects of JT distortions—for example the precise type of orbital order/disorder or the identity of the JT-active cation—and to what extent they couple to the properties. Our focus has been on Cu^II^, but Mn^III^ is common in both oxides^90^ and in some PBAs;^91^ and the pressure behaviour of a JT-distorted nickelate was recently reported.^92^ At any rate, the clear structural and functional implications of JT distortions—as demonstrated here and elsewhere—highlight the clear potential for JT distortions to act as a crystal engineering element for the development of interesting functionality.

## Data availability

Data for this paper, including raw XRD and ND data, are available at DOI: 10.58141/514y-vb19 and at DOI: 10.5286/ISIS.E.RB2220172. Crystal structures for RbCuCo and CsCuCo can be found in the ICSD database under the deposition numbers CSD 2325814–2325815.

## Author contributions

HLBB designed the study and all authors contributed to the data collection. HLBB analysed and interpreted the data with assistance from ABC and NPF. HLBB wrote the paper with input from all other authors.

## Conflicts of interest

There are no conflicts to declare.

## Supplementary Material

SC-015-D3SC06912E-s001

SC-015-D3SC06912E-s002

SC-015-D3SC06912E-s003

SC-015-D3SC06912E-s004
