# Supplementary material for: The pressure response of Jahn–Teller-distorted Prussian blue analogues
Source: Chem Sci. 2024 Jan 23;15(9):3155–64. doi: 10.1039/d3sc06912e (PMC10901509; doi:10.1039/d3sc06912e)
Supplement: SC-015-D3SC06912E-s004 [file SC-015-D3SC06912E-s004.pdf]

Electronic Supplementary Material (ESI) for Chemical Science.
This journal is © The Royal Society of Chemistry 2024

The pressure response of Jahn-Teller distorted Prussian blue analogues
SUPPORTING INFORMATION

Hanna L. B. Boström,[a,b,c*] Andrew B. Cairns,[d,e] Muzi Chen,[d,e]
Dominik Daisenberger,[f] Christopher J. Ridley,[g] and Nicholas P. Funnell[g]

[a] Max Planck Institute for Solid State Research, Heisenbergstraße 1,
D-70569 Stuttgart, Germany.
[b] Division of Materials and Environmental Chemistry, Stockholm University,
Svante Arrhenius väg 16C, SE-106 91 Stockholm, Sweden.
[c] Wallenberg Initiative Materials Science for Sustainability, Department of Materials
and Environmental Chemistry, Stockholm University, SE-114 18 Stockholm, Sweden.
[d] Department of Materials, Imperial College London, Royal School of Mines,
Exhibition Road, London SW7 2AZ, U.K.
[e] London Centre for Nanotechnology, Imperial College London, London SW7 2AZ, U.K.
[f] Diamond Light Source Ltd., Harwell Campus, Didcot OX11 0DE, U.K.
Harwell Campus, Didcot OX11 0DE, UK
[g] ISIS Neutron and Muon Source, Rutherford Appleton Laboratory,
Harwell Campus, Didcot OX11 0QX, U.K.

*To whom correspondence should be addressed;
E-mail: hanna.bostrom@mmk.su.se

Contents

1 Experimental details **3**

2 Variable-pressure diffraction patterns **5**

3 Rietveld and Pawley fits **13**

4 Variable-pressure lattice parameters **22**

5 Birch–Murnaghan fits **31**

6 References **36**

1 Experimental details

The synthesis and compositional characterisation of RbCuCo are described in Ref. 1. Caution: the metals used are harmful to health and all work with cyanide-containing compounds should be performed in an area free from acids, to prevent the accidental release of highly toxic HCN gas. $Cu[Co]_{0.67}$ was synthesised by adding $K_3Co(CN)_6$ (111 mg, 0.33 mmol, 1 ml H_2O) to an aqueous solution of $CuCl_2{\cdot}2H_2O$ (88.7 mg, 0.53 mmol, 1 ml H_2O). After stirring for 1 h at 50 °C and 1 h at room temperature, the blue product was isolated by centrifugation, washed with 2×4 ml of water and dried in air. CuPt was synthesised by the slow addition of an aqueous solution of $CuSO_4$ (192.5 mg, 1.20 mmol, 3 ml H_2O) to an aqueous solution of $K_2Pt(CN)_6$ (517.5 mg, 1.20 mmol, 1.5 ml H_2O) and stirred at 60 ° for 1 h. The product was isolated as a blue powder by filtration and dried first in air and then in vacuum overnight. CsCuCo was synthesised by slowly adding $CuCl_2{\cdot}2H_2O$ (51.1 mg, 0.3 mmol, 0.5 ml H_2O) to an aqueous solution of CsCl ($\sim$ 1.7 g) and $K_3Co(CN)_6$ (99.7 mg, 0.3 mmol, 2.5 ml H_2O). The reaction mixture was stirred for 2 h at 50 °C. The pale green-blue solution was centrifuged, washed with at least 2 × 4 ml of water, and dried in air.

For CsCuCo and $Cu[Co]_{0.67}$, elemental analysis was carried out by a Varian Vista-PRO ICP-OES spectrometer with axial plasma (Fa. Variant Darmstadt). The samples were dissolved in 65% HNO_3 at 185 °C for 25 min *via* microwave digestion using a Discover SP-D from CEM GmbH and then diluted with doubly distilled water. Caution: this step must be carried out in a well-ventilated fumehood using only small amounts of sample (a few mg) to avoid HCN exposure.

Variable-pressure powder X-ray diffraction was performed on beamline I15 at Diamond Light Source, at an energy of 33 keV. Polycrystalline samples were loaded into a diamond anvil cell (DAC), with Daphne 7373 oil as a pressure-transmitting medium and a ruby for pressure calibration. All samples—except $Cu[Co]_{0.67} \cdot nH_2O$ and $CuPt{\cdot}nH_2O$—were evacuated at 80 °C under vacuum overnight prior to the beamtime. $Cu[Co]_{0.67}$, RbCuCo and CuPt were immediately transferred to a glovebox to prevent rehydration and loading of these samples was likewise carried out in a glovebox. To minimise radiation damage, the beam intensity was attenuated with aluminium plates. The sample-to-detector distance was calibrated following standard procedures from the diffraction ring of a LaB_6 standard. The best-scattering sample position of the cell was measured at every pressure point, and to check for beam damage, data from other sample positions were periodically sampled. The data were processed using Dioptas.[S2]

Variable-pressure neutron powder diffraction was carried out on the PEARL diffractometer[S3] at the ISIS Neutron and Muon Source, U.K. The ground powder was loaded in a null-scattering TiZr gasket along with a Pb pressure marker, and placed between single-toroid ZrO_2-toughened

Al_2O_3 anvils. The pressure-transmitting medium was a perdeuterated methanol–ethanol mixture in a 4:1 volume ratio. The anvil assembly was then loaded in a V3 Paris-Edinburgh press and mounted in the PEARL instrument. Pressure was controlled *via* an oil-driven piston. The data were processed using Mantid.[S4]

To obtain lattice parameters from the XRD data, the patterns were refined by sequential Pawley refinements in the software TOPAS.[S5] The ND patterns were refined by Rietveld and Pawley (RbCuCo) and only Pawley (CsCuCo) refinements, respectively. The PEARL diffractometer has the capability of measuring longer-wavelength neutrons due to the division of proton pulses between the target stations at ISIS, so that it can access longer d-spacings. However, this is only possible for one pulse in four, which leads to a corresponding reduction in measurement statistics. Therefore only Pawley fits were performed against data collected from long-wavelength neutrons. Peakshape parameters, lattice parameters and background terms were refined.[S5–7] For the neutron data, the peak shape was modelled as a pseudo-Voigt function convoluted with a back-to-back double exponential and the modified Thompson-Cox-Hastings pseudo-Voigt was used for the I15 data. To account for the anisotropic peak shapes caused by Cs disorder in CsCuCo, an additional Lorentzian *hkl*-dependent peak broadening term was added to the refinement of the X-ray data, whereas a sample-dependent time-of-flight peakshape was used for the ND data.

Rietveld refinements of diffraction patterns at ambient conditions of CuPt, CsCuCo, and RbCuCo were carried out *via* symmetry-mode refinements, as implemented in ISODISTORT.[S8] For the latter sample, distortion modes corresponding to symmetric strain (Γ_1^+), octahedral tilting (X_3^+), and Jahn-Teller distortion (Γ_3^+) were refined. Likewise, only the Jahn-Teller distortion (Γ_3^+) was refined for CuPt and CsCuCo. The CN bond lengths were restrained to 1.14 Å in all cases. For the ND refinement of RbCuCo, the Rb distribution over the two independent sites was refined against the XRD data and fixed during the ND refinements. The structure for the Rietveld fit of $Cu[Co]_{0.67}$ was taken from Ref. 9 and no positional parameters were refined.

The lattice parameters as a function of pressure were extracted using Pawley refinements. The variable-pressure unit cell lattice parameters were fitted using a second-order Birch–Murnaghan equation of state as implemented in the software EoSfit-GUI.[S10–12] For the XRD data, only pressure points up to 2.2 GPa were used—as the pressure-transmitting medium is no longer hydrostatic beyond this point[S13]—and only data from the best-scattering sample position. Linear compressibilities were calculated by PASCal.[S14]

2 Variable-pressure diffraction patterns

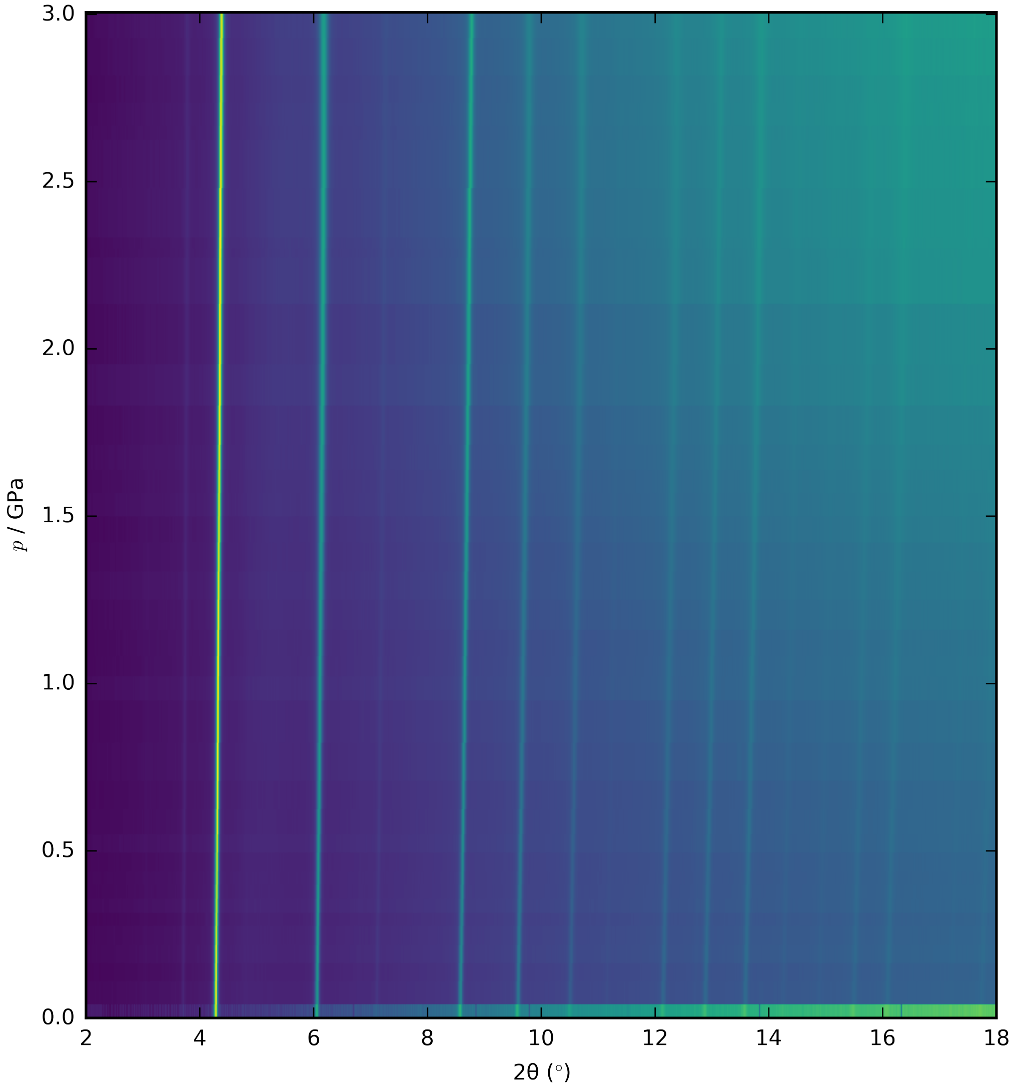

Figure S1: The XRD patterns of $Cu[Co]_{0.67} \cdot nH_2O$ under variable pressure collected at I15, Diamond Light Source. The intensity of the most intense reflection is normalised to 1. $\lambda = 0.3757$ Å.

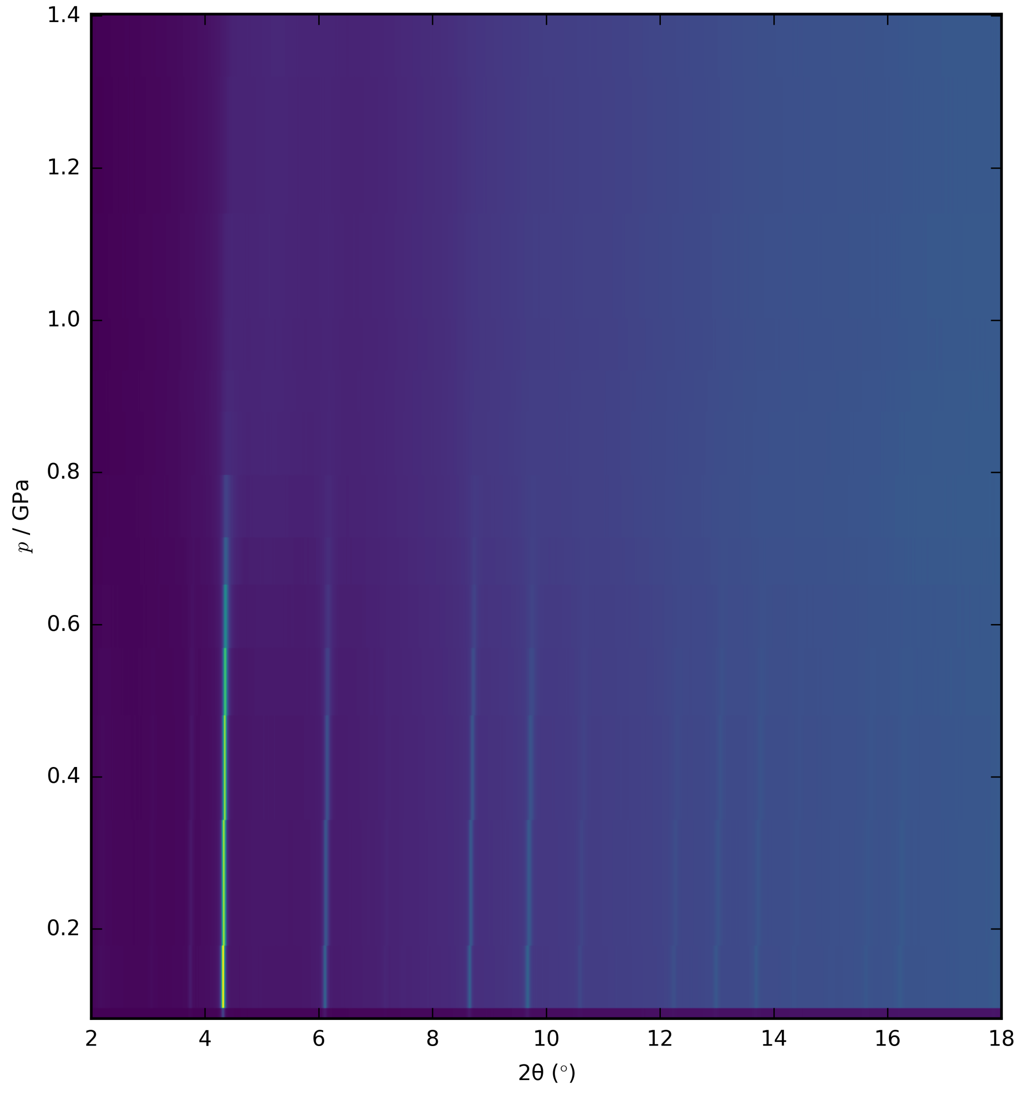

Figure S2: The XRD patterns of Cu[Co]$_{0.67}$ under variable pressure collected at I15, Diamond Light Source. $\lambda = 0.3757\,\text{Å}$.

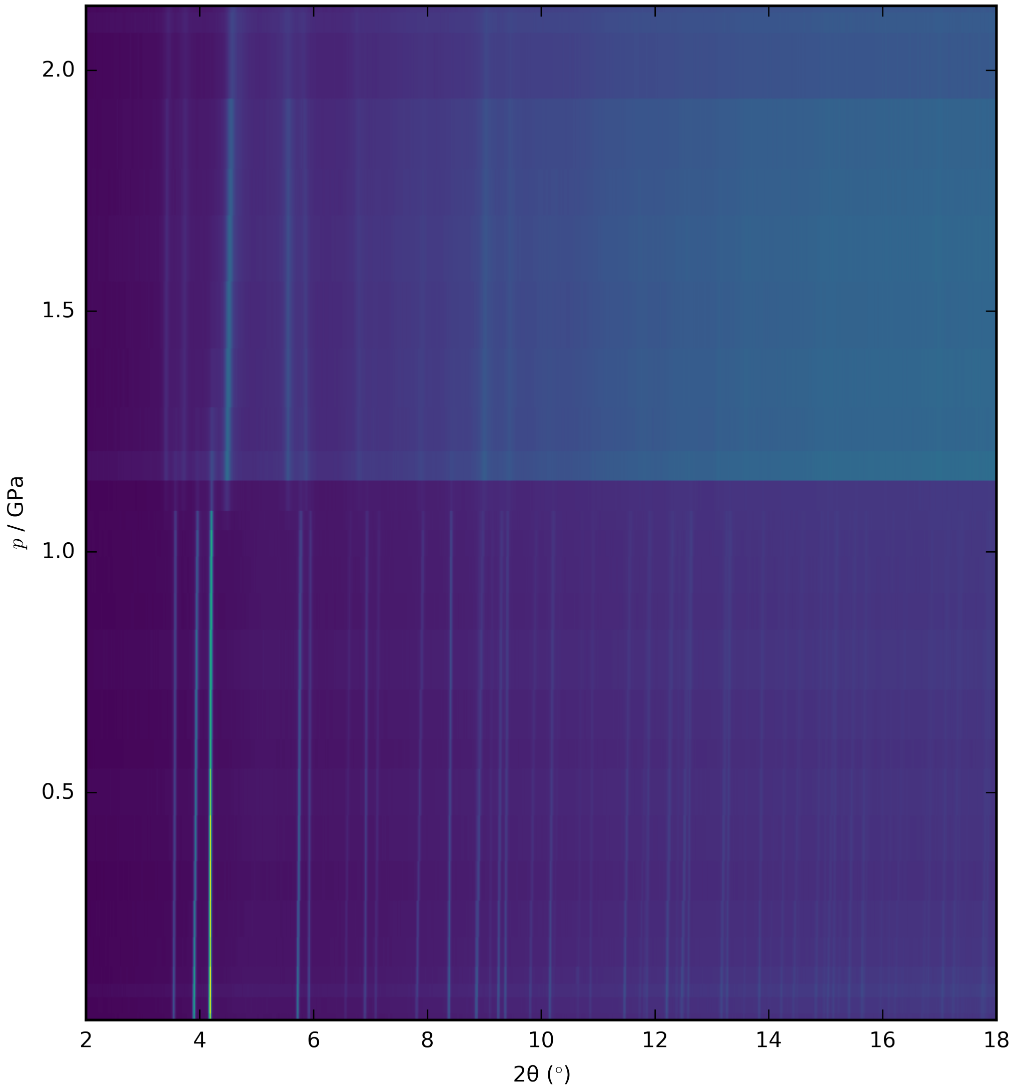

Figure S3: The XRD patterns of CuPt·nH$_2$O under variable pressure collected at I15, Diamond Light Source. The intensity increase at ~ 1.2 GPa is due to an increase in collection time. $\lambda = 0.3757$ Å.

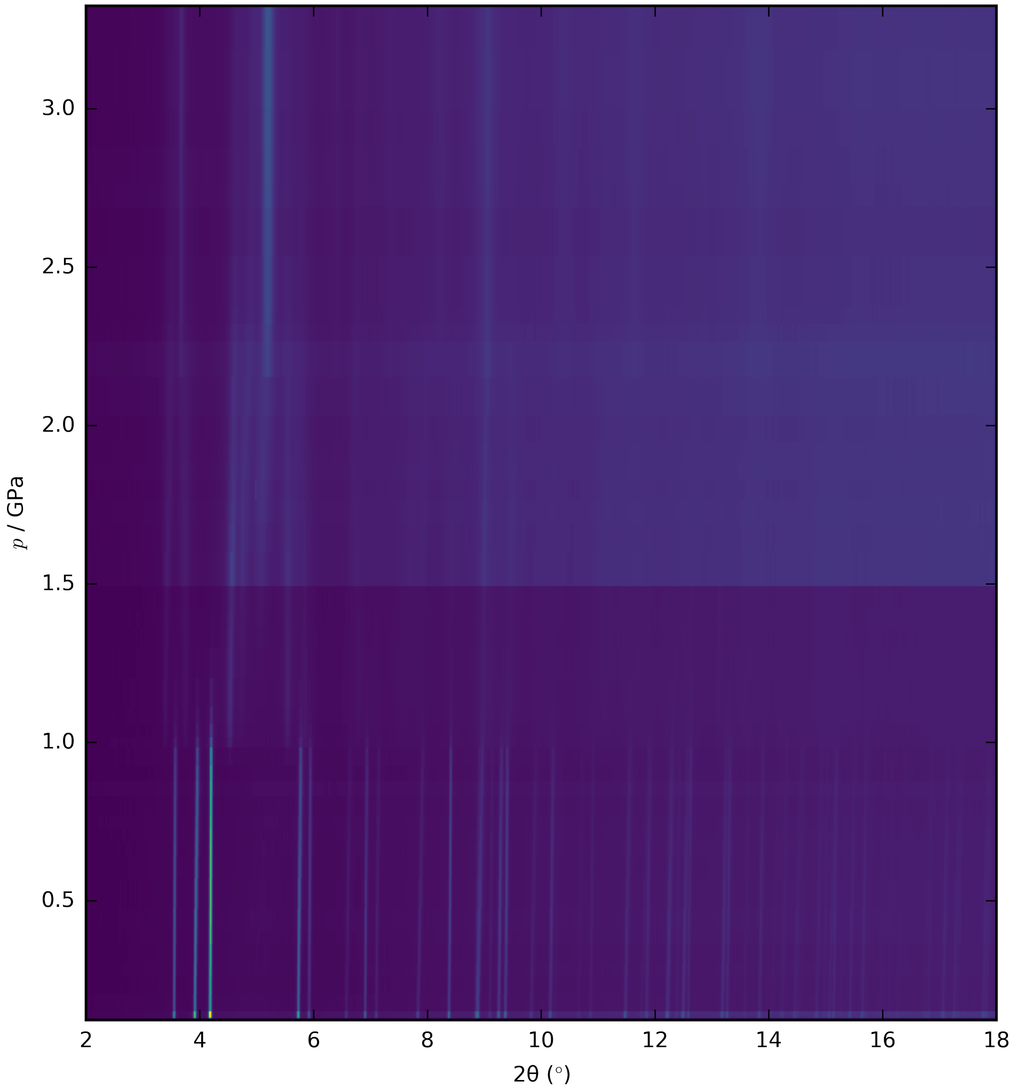

Figure S4: The XRD patterns of CuPt under variable pressure collected at I15, Diamond Light Source. The intensity increase at $\sim 1.5\,$GPa is due to an increase in collection time. $\lambda = 0.3757\,$Å.

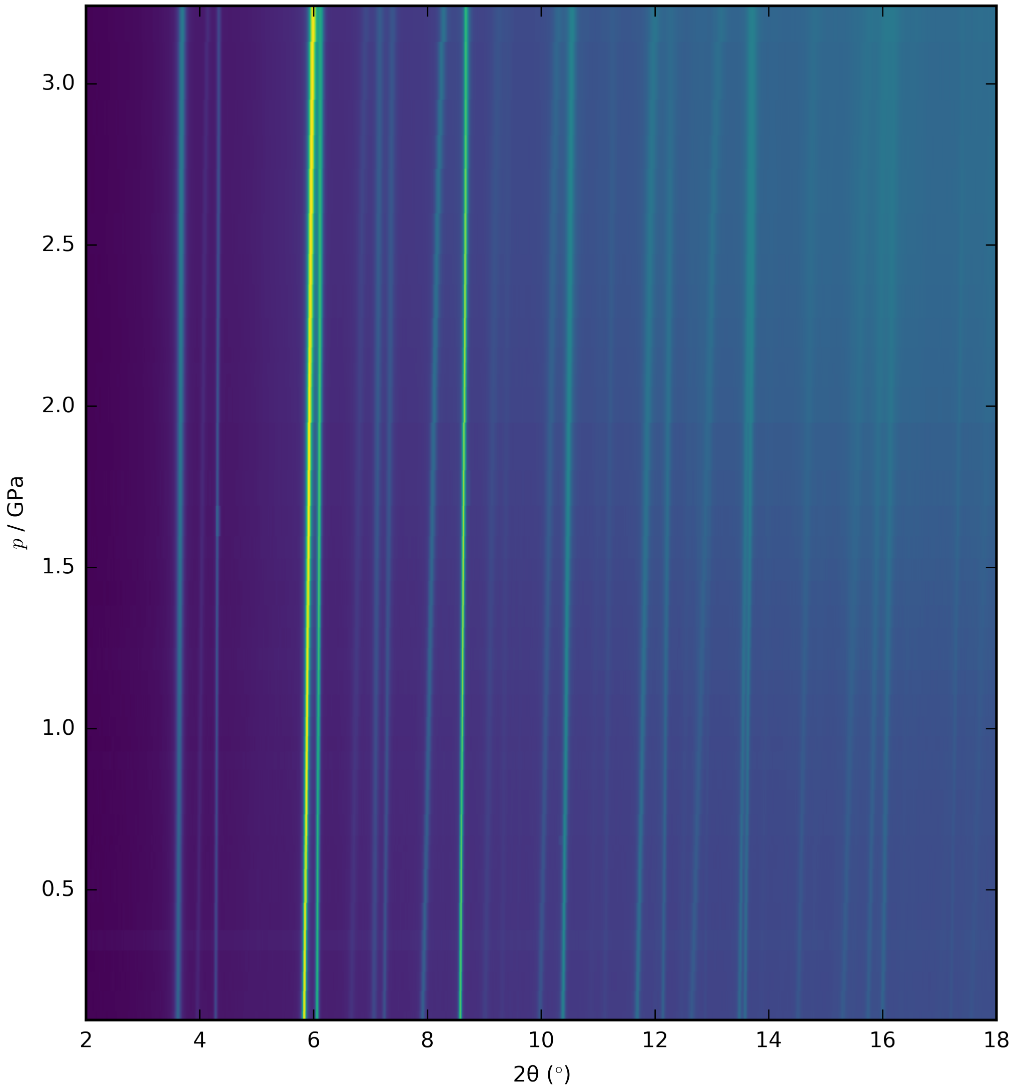

Figure S5: The XRD patterns of CsCuCo under variable pressure collected at I15, Diamond Light Source. The intensitiy of the most intense reflection is normalised to 1. $\lambda = 0.3757\,\text{Å}$.

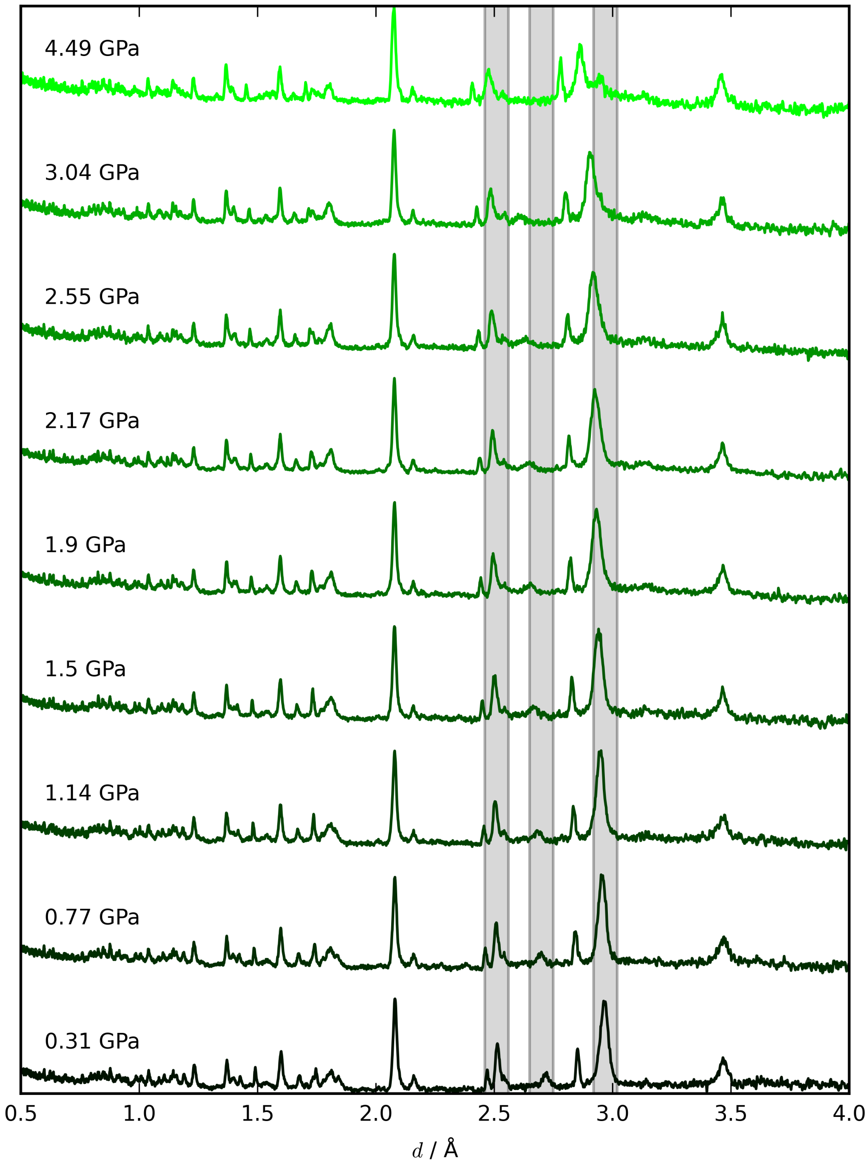

Figure S6: The ND data of CsCuCo under variable pressure collected at PEARL, ISIS Muon and Neutron Source. Grey areas mark areas with reflections from the sample at 0 GPa.

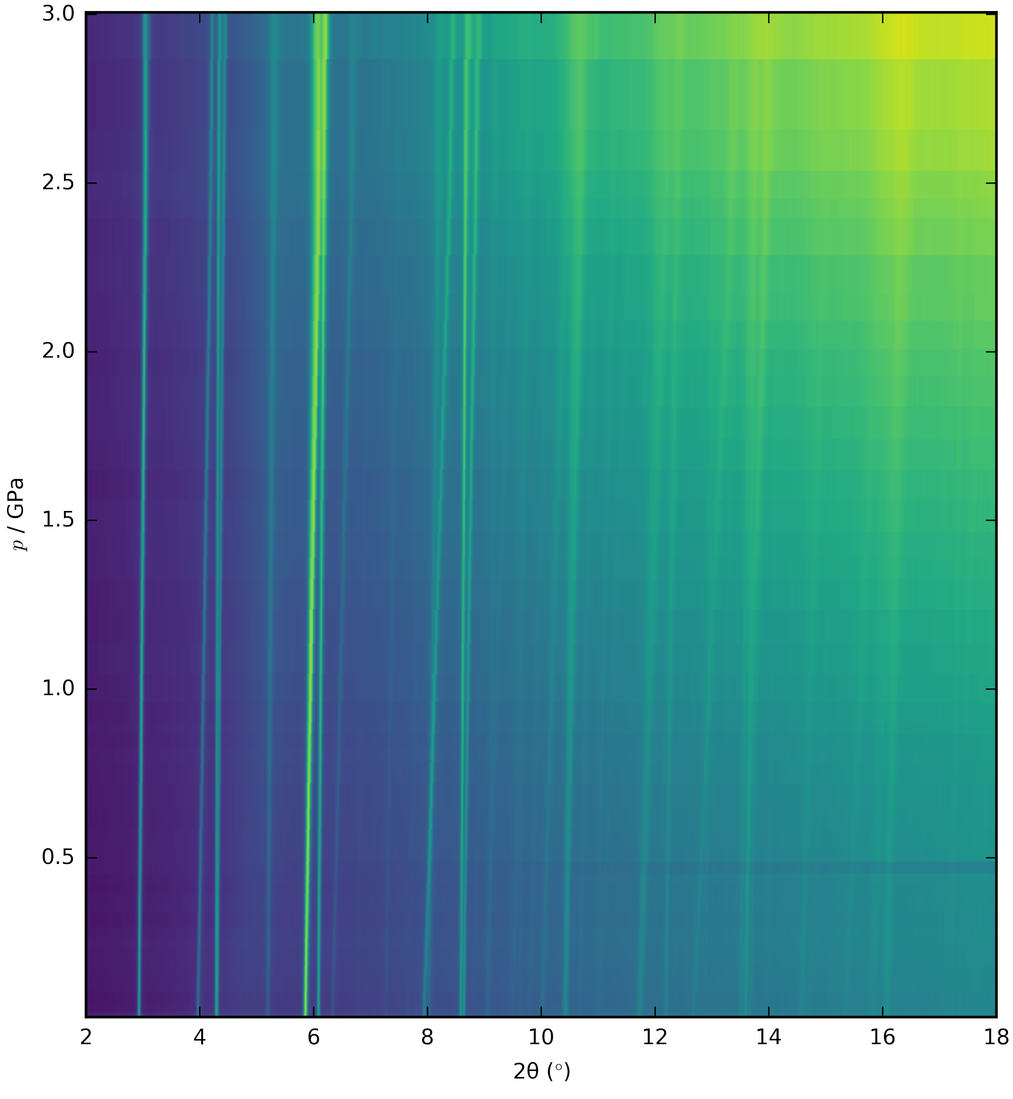

Figure S7: The XRD patterns of RbCuCo under variable pressure collected at I15, Diamond Light Source. The intensity of the most intense reflection is normalised to 1. $\lambda = 0.3757$ Å.

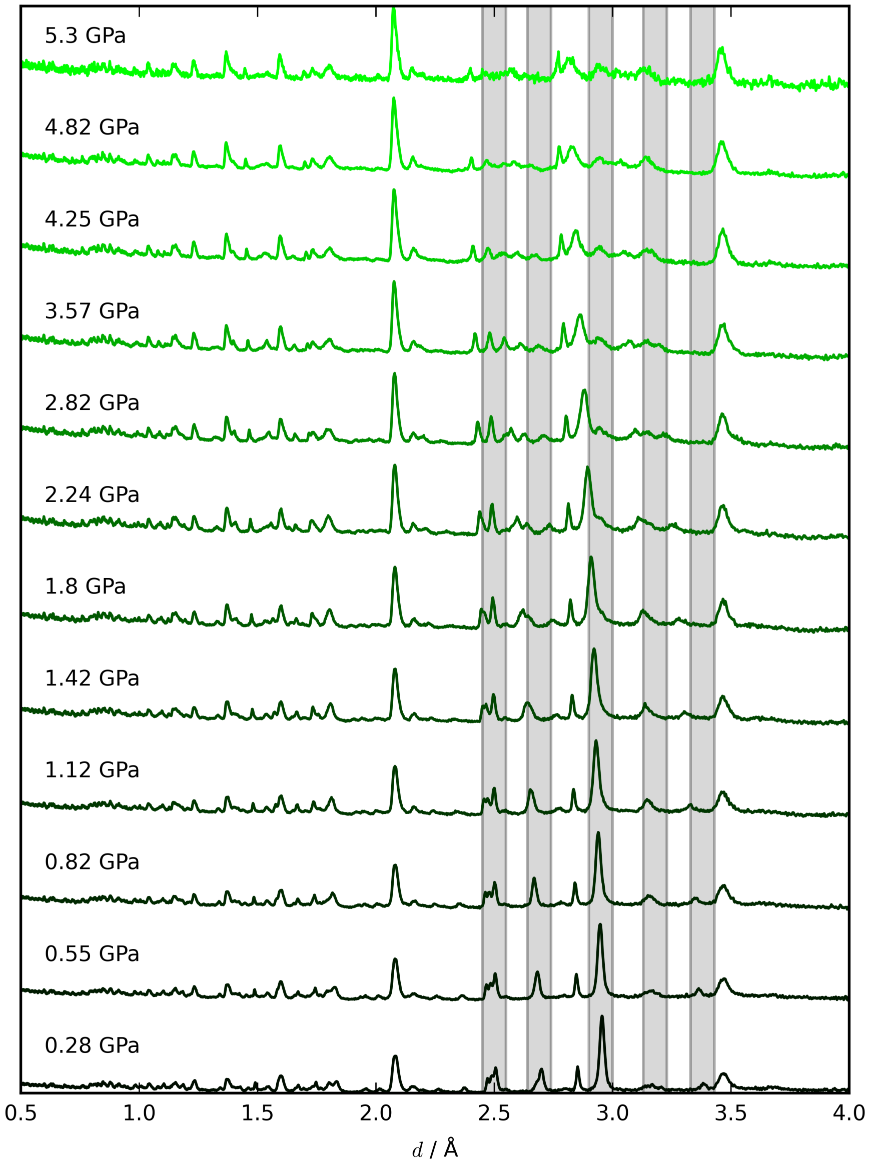

Figure S8: The ND data of RbCuCo under variable pressure collected at PEARL, ISIS Muon and Neutron Source. Grey areas mark areas with reflections from the sample at 0 GPa.

3 Rietveld and Pawley fits

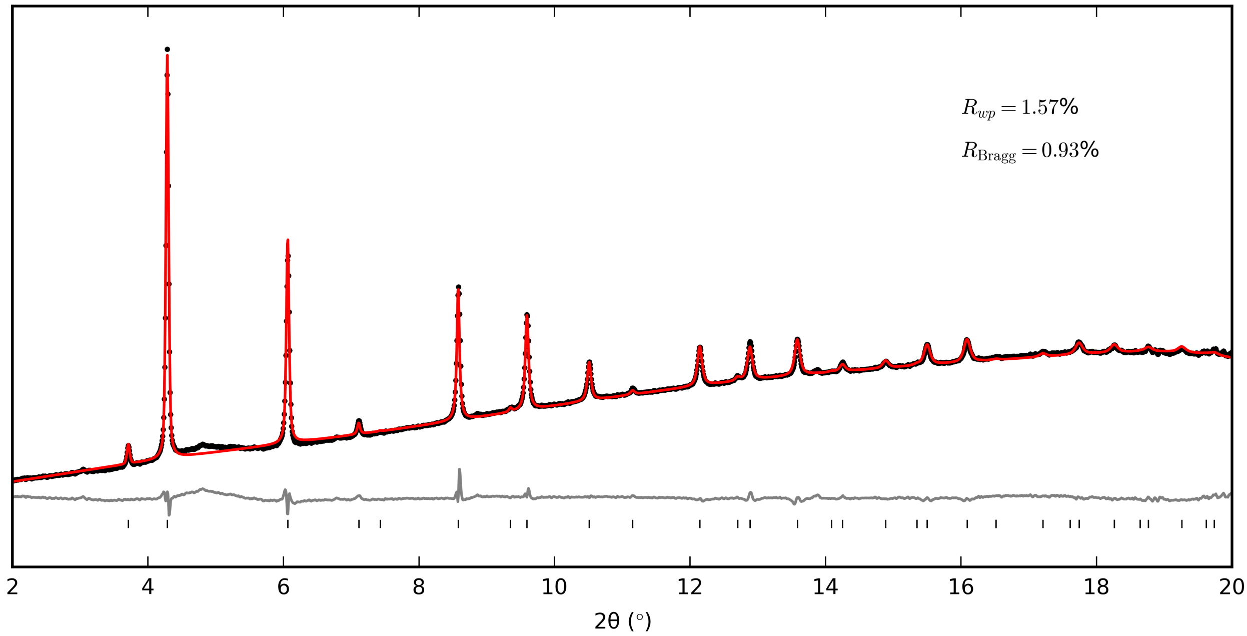

Figure S9: The Rietveld fit of the ambient phase of Cu[Co]$_{0.67} \cdot n$H$_2$O in $Fm\bar{3}m$ at 0.08 GPa collected at I15, Diamond Light Source. Experimental data are shown in black, the fit in red, the residuals in grey, and allowed reflections are indicated by vertical tickmarks.

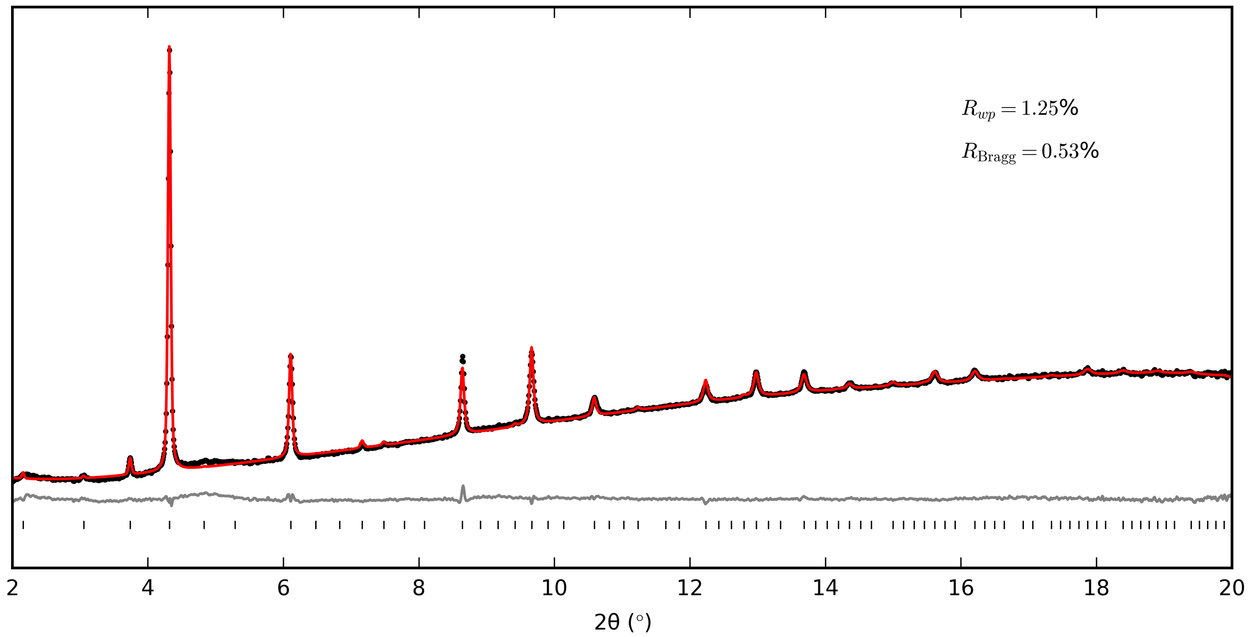

Figure S10: The Rietveld fit of the ambient phase of Cu[Co]$_{0.67}$ in $Pm\bar{3}m$ at 0.08 GPa collected at I15, Diamond Light Source. Experimental data are shown in black, the fit in red, the residuals in grey, and allowed reflections are indicated by vertical tickmarks.

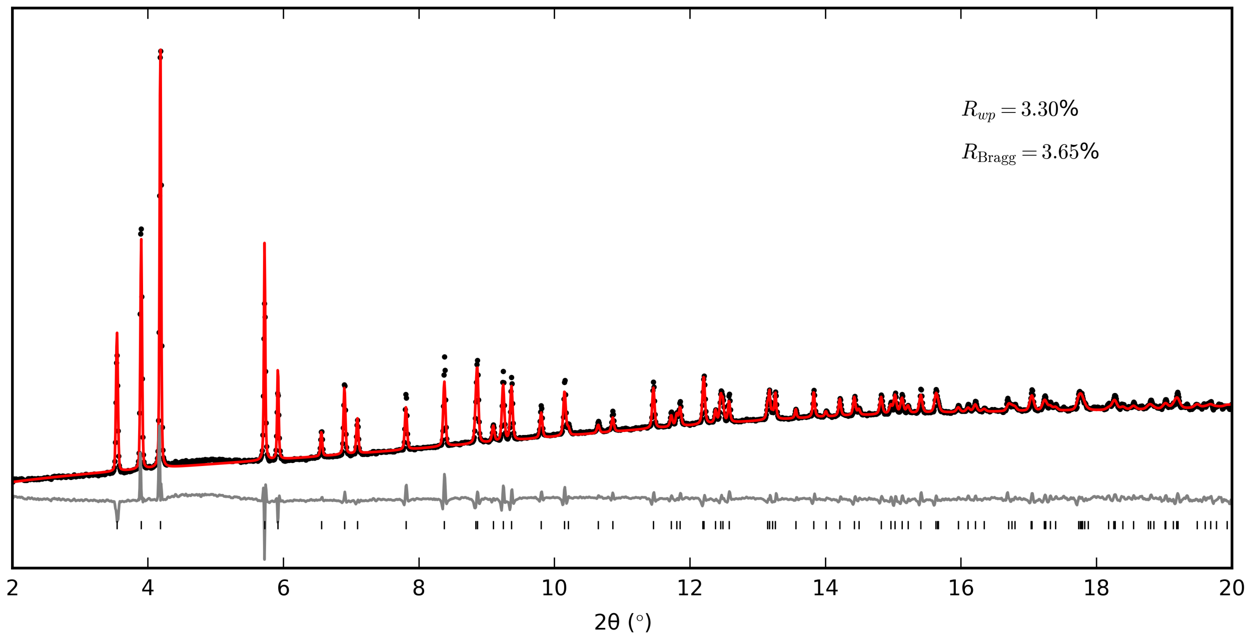

Figure S11: The Rietveld fit of the ambient phase of CuPt$\cdot n$H$_2$O in $I4/mmm$ at 0.03 GPa collected at I15, Diamond Light Source. Experimental data are shown in black, the fit in red, the residuals in grey, and allowed reflections are indicated by vertical tickmarks.

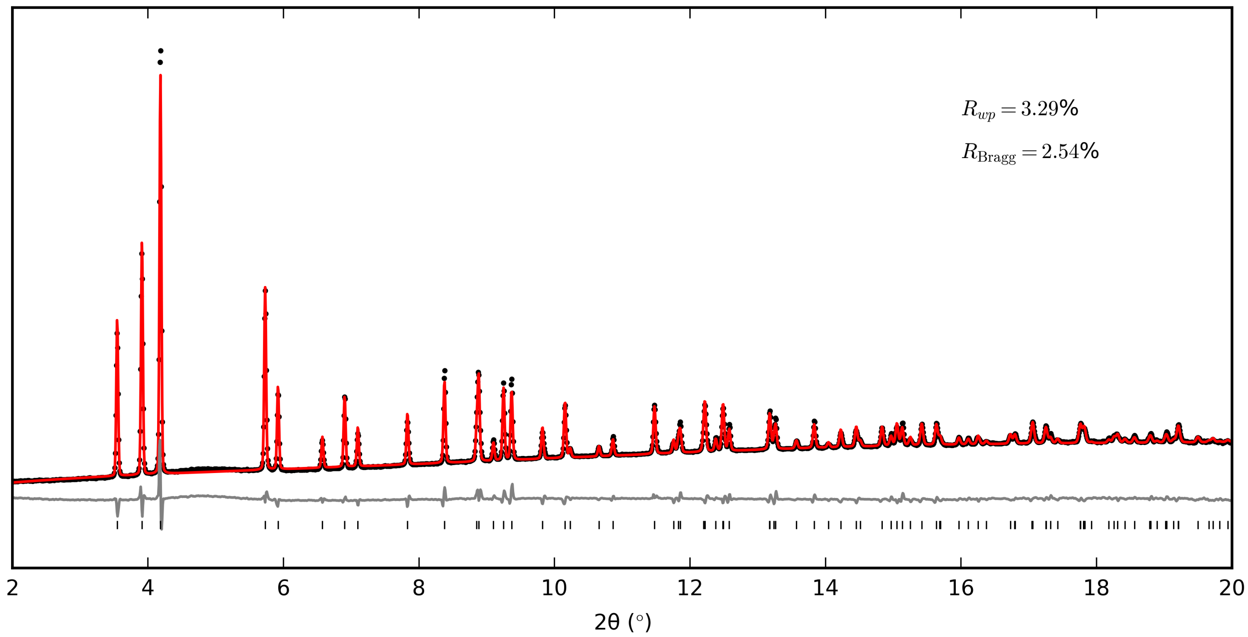

Figure S12: The Rietveld fit of the ambient phase of CuPt in $I4/mmm$ at 0.14 GPa collected at I15, Diamond Light Source. Experimental data are shown in black, the fit in red, the residuals in grey, and allowed reflections are indicated by vertical tickmarks.

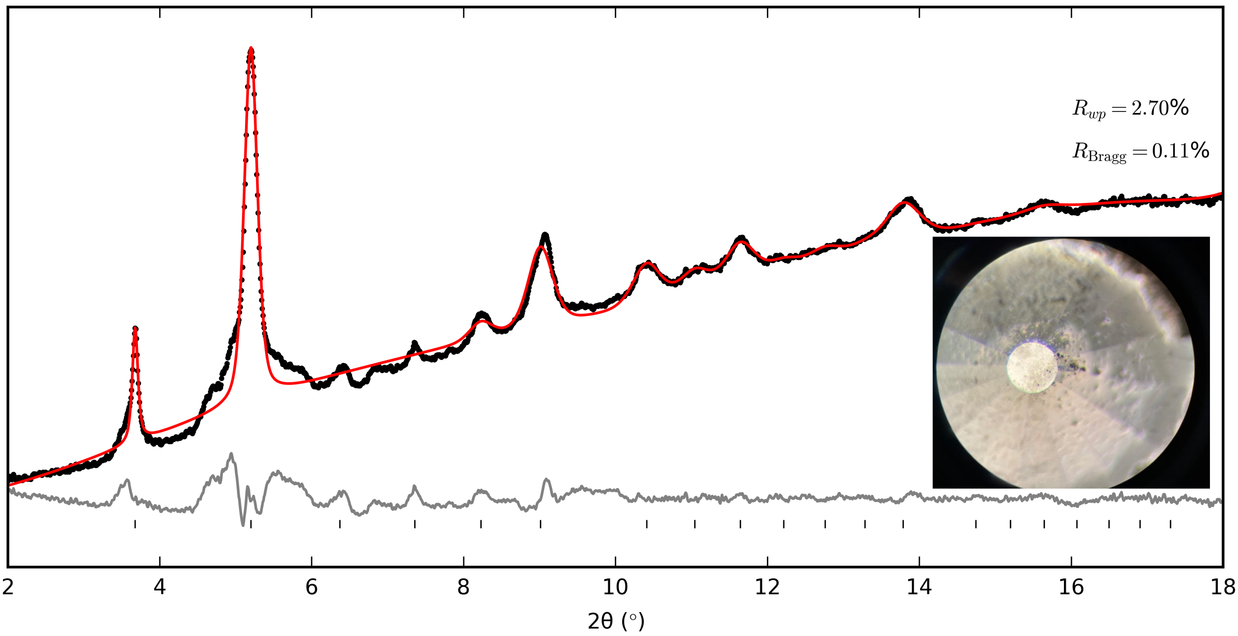

Figure S13: The Pawley fit of the cubic high-pressure phase of CuPt in $Pm\bar{3}m$ at 3.32 GPa collected at I15, Diamond Light Source. Experimental data are shown in black, the fit in red, the residuals in grey, and allowed reflections are indicated by vertical tickmarks. The inset shows a photo of the DAC following decompression.

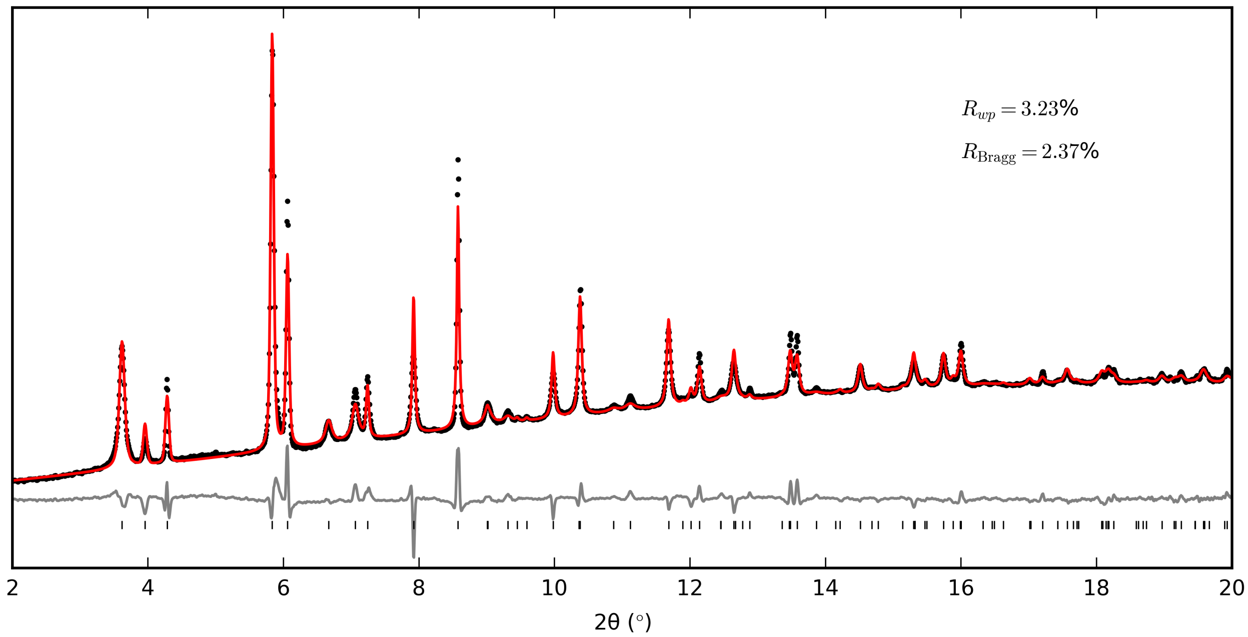

Figure S14: The Rietveld fit of the ambient phase of CsCuCo in $I\bar{4}m2$ at 0.11 GPa collected at I15, Diamond Light Source. Experimental data are shown in black, the fit in red, the residuals in grey, and allowed reflections are indicated by vertical tickmarks.

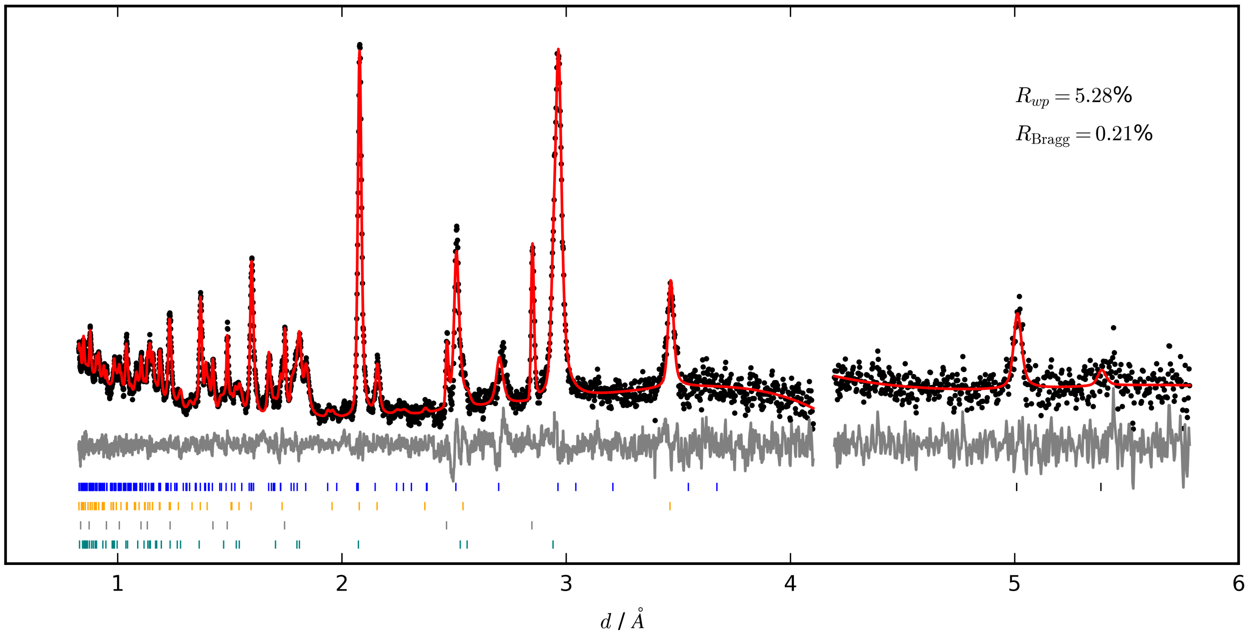

Figure S15: The Pawley fit of the ambient phase of CsCuCo in $I\bar{4}m2$ at 0.308(9) GPa collected at PEARL, ISIS Neutron and Muon Source. Experimental data are shown in black, the fit in red, the residuals in grey, and allowed reflections are indicated by vertical tickmarks. Colour code: CsCuCo (blue), alumina (yellow), lead (grey), and zirconia (teal).

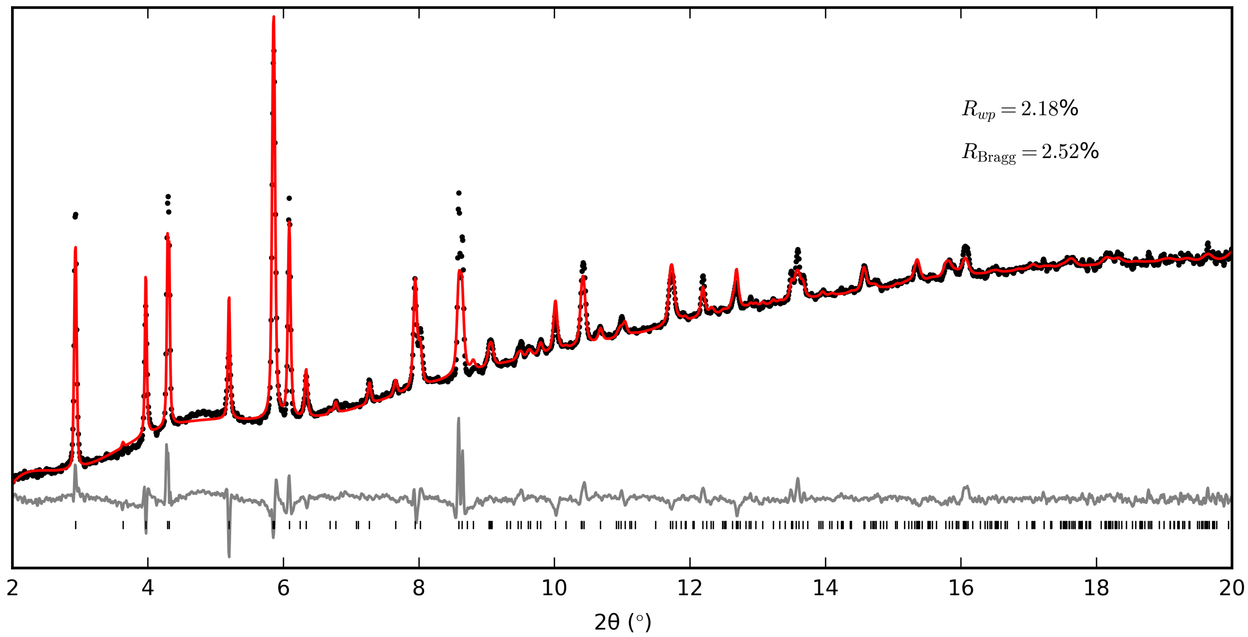

Figure S16: The Rietveld fit of the ambient phase of RbCuCo in $Cccm$ at 0.03 GPa collected at I15, Diamond Light Source. Experimental data are shown in black, the fit in red, the residuals in grey, and allowed reflections are indicated by vertical tickmarks.

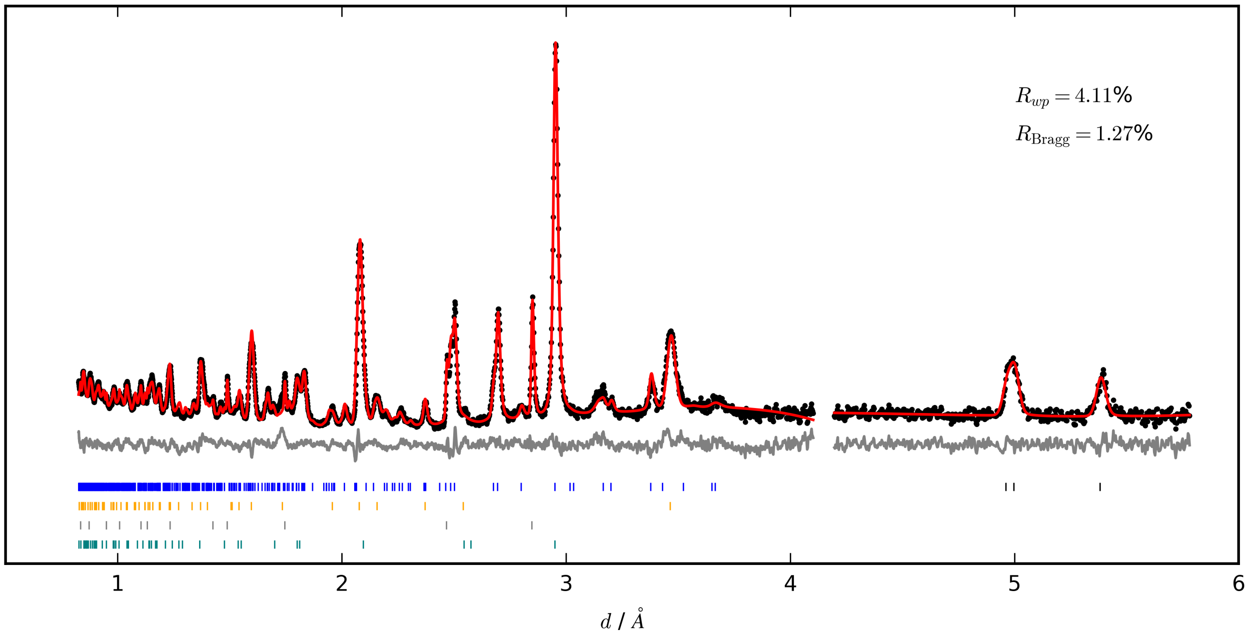

Figure S17: The Rietveld fit of the ambient phase of RbCuCo in $Cccm$ at 0.282(4) GPa collected at PEARL, ISIS Neutron and Muon Source. Experimental data are shown in black, the fit in red, the residuals in grey, and allowed reflections are indicated by vertical tickmarks. Colour code: RbCuCo (blue), alumina (yellow), lead (grey), and zirconia (teal).

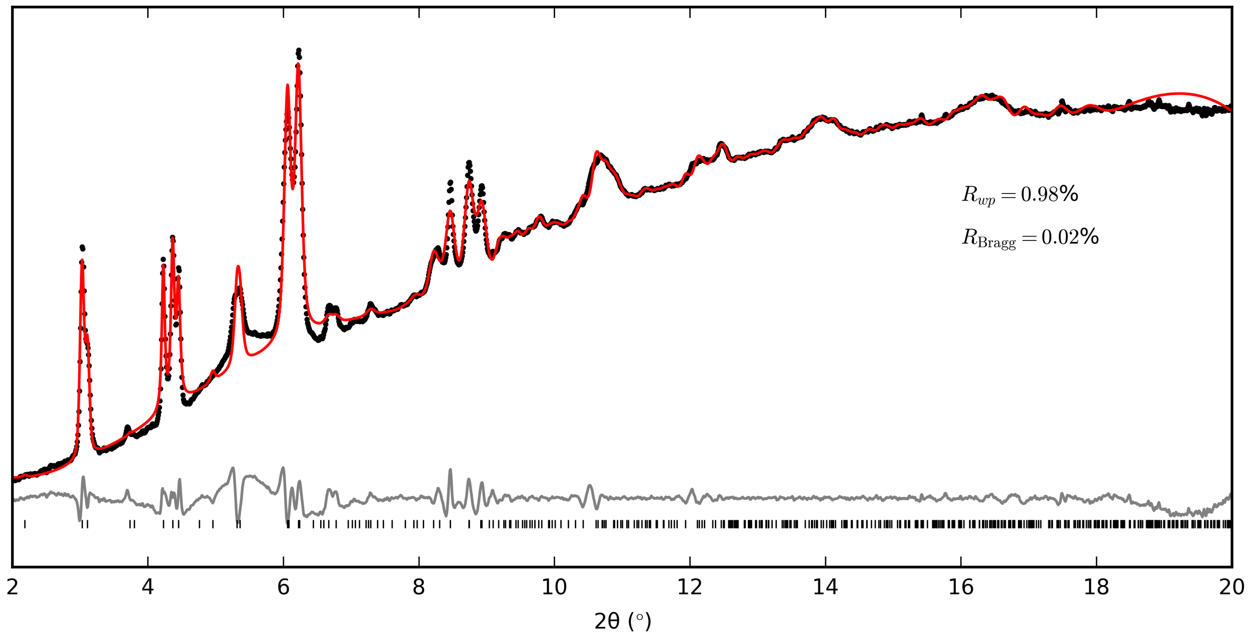

Figure S18: The Pawley fit of the ambient phase of RbCuCo in $P2/m$ ($a = 6.93$ Å, $b = 9.87$ Å, $c = 7.11$ Å, $\beta = 87.1\,°$) at 3.04 GPa collected at I15, Diamond Light Source. Experimental data are shown in black, the fit in red, the residuals in grey, and allowed reflections are indicated by vertical tickmarks.

4 Variable-pressure lattice parameters

Table S1: Variable-pressure lattice parameters of $Cu[Co]_{0.67} \cdot nH_2O$ in $Fm\bar{3}m$ collected at I15, Diamond Light Source. Uncertainty is ± 0.1 GPa at all pressures.

p / GPa	a / Å	V / Å^3
0.00	10.0581(3)	1017.54(7)
0.00	10.0582(2)	1017.58(6)
0.00	10.0449(2)	1013.52(6)
0.08	10.0444(2)	1013.38(5)
0.14	10.0384(2)	1011.58(6)
0.19	10.0321(2)	1009.67(6)
0.25	10.0275(2)	1008.27(6)
0.30	10.0227(2)	1006.82(6)
0.33	10.0180(2)	1005.42(6)
0.38	10.0142(2)	1004.27(6)
0.41	10.0102(2)	1003.05(6)
0.47	10.0073(2)	1002.18(6)
0.52	10.0010(2)	1000.31(6)
0.58	9.9949(2)	998.48(6)
0.67	9.9853(2)	995.59(6)
0.74	9.9797(2)	993.92(7)
0.91	9.9677(3)	990.34(7)
0.99	9.9617(3)	988.56(7)
1.05	9.9581(3)	987.48(7)
1.11	9.9520(3)	985.67(7)
1.21	9.9427(3)	982.89(8)
1.29	9.9354(3)	980.74(8)
1.37	9.9291(3)	978.88(8)
1.47	9.9217(3)	976.70(8)
1.53	9.9190(3)	975.91(8)
1.61	9.9147(3)	974.63(8)
1.66	9.9089(3)	972.92(9)
1.76	9.9013(3)	970.68(9)

Table S1: Variable-pressure lattice parameters of Cu[Co]$_{0.67}$·nH$_2$O in $Fm\bar{3}m$ collected at I15, Diamond Light Source. Uncertainty is ± 0.1 GPa at all pressures.

p / GPa	a / Å	V / Å^3
1.90	9.8896(3)	967.23(9)
2.01	9.8814(3)	964.84(9)

Table S2: Variable-pressure lattice parameters of Cu[Co]$_{0.67}$ in $Pm\bar{3}m$ collected at I15, Diamond Light Source. Uncertainty is ± 0.1 GPa at all pressures.

p / GPa	a / Å	V / Å^3
0.08	9.9729(2)	991.88(6)
0.11	9.9691(2)	990.76(6)
0.11	9.9678(2)	990.37(5)
0.11	9.9659(2)	989.82(6)
0.25	9.9379(2)	981.50(6)
0.44	9.9048(3)	971.70(7)
0.52	9.8838(4)	965.54(11)
0.62	9.8629(7)	959.4(2)
0.69	9.841(2)	953.1(5)

Table S3: Variable-pressure lattice parameters of CuPt·nH$_2$O in $I4/mmm$ collected at I15, Diamond Light Source. Uncertainty is ± 0.1 GPa at all pressures.

p / GPa	a / Å	c / Å	V / Å^3
0.03	7.27573(12)	11.0352(4)	584.16(3)
0.05	7.27547(12)	11.0337(4)	584.04(3)
0.10	7.27393(11)	11.0267(4)	583.42(3)
0.11	7.27277(11)	11.0191(4)	582.83(3)
0.16	7.27107(11)	11.0122(4)	582.19(3)
0.23	7.26975(12)	11.0040(4)	581.56(3)
0.32	7.26688(14)	10.9829(4)	579.98(3)
0.40	7.26437(14)	10.9690(4)	578.84(4)
0.51	7.26102(14)	10.9534(4)	577.49(3)

Table S3: Variable-pressure lattice parameters of CuPt·nH$_2$O in $I4/mmm$ collected at I15, Diamond Light Source. Uncertainty is ± 0.1 GPa at all pressures.

p / GPa	a / Å	c / Å	V / Å^3
0.59	7.25744(14)	10.9411(4)	576.27(3)
0.63	7.25535(14)	10.9309(4)	575.41(3)
0.80	7.25098(14)	10.9093(5)	573.58(4)
0.88	7.24774(14)	10.8962(5)	572.37(4)
0.95	7.24534(14)	10.8863(5)	571.47(4)
1.03	7.2408(2)	10.8766(5)	570.24(4)

Table S4: Variable-pressure lattice parameters of CuPt in $I4/mmm$ collected at I15, Diamond Light Source. Uncertainty is ± 0.1 GPa at all pressures.

p / GPa	a / Å	c / Å	V / Å^3
0.12	7.27365(12)	11.0028(4)	582.11(3)
0.14	7.27398(12)	11.0038(4)	582.22(3)
0.16	7.27180(12)	10.9937(4)	581.34(3)
0.25	7.26990(12)	10.9846(4)	580.55(3)
0.25	7.26932(12)	10.9827(4)	580.36(3)
0.27	7.26802(12)	10.9763(4)	579.81(3)
0.33	7.26615(12)	10.9679(4)	579.07(3)
0.40	7.26401(14)	10.9594(4)	578.28(3)
0.41	7.26348(14)	10.9570(4)	578.07(3)
0.43	7.26244(14)	10.9530(4)	577.70(3)
0.52	7.25936(14)	10.9387(4)	576.45(3)
0.62	7.25652(14)	10.9259(5)	575.33(4)
0.66	7.25458(15)	10.9169(5)	574.54(4)
0.71	7.25300(15)	10.9101(5)	573.94(4)
0.74	7.2519(2)	10.9057(5)	573.54(4)
0.80	7.2499(2)	10.8977(5)	572.80(4)
0.87	7.2478(2)	10.8878(5)	571.94(4)
0.89	7.2465(2)	10.8820(5)	571.43(4)

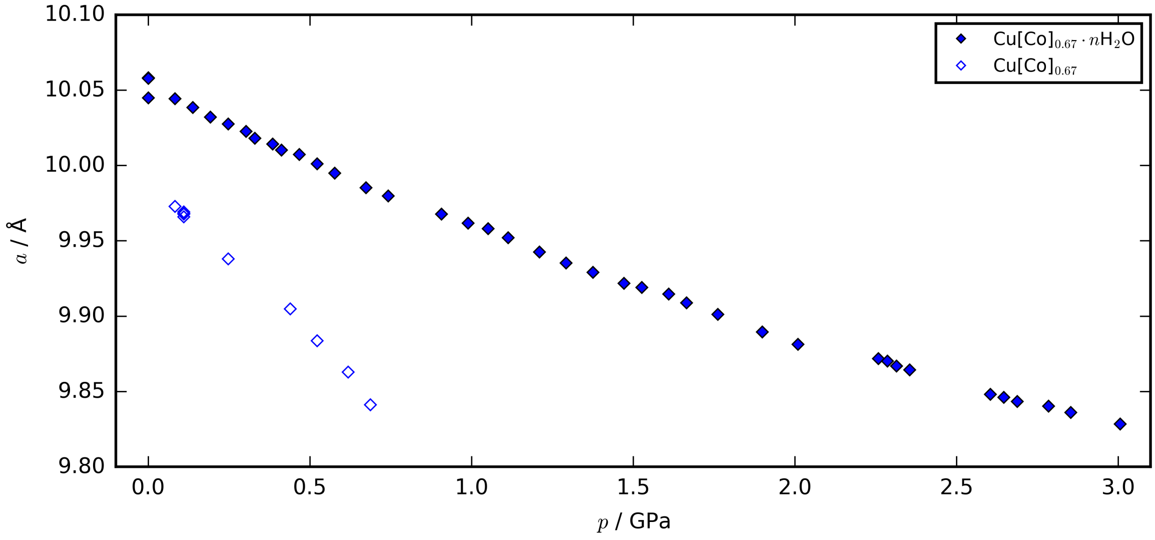

Figure S19: The evolution of the lattice parameters of Cu[Co]$_{0.67}$ and Cu[Co]$_{0.67} \cdot n$H$_2$O as a function of pressure. Error bars are smaller than the data markers and therefore omitted.

Table S5: Variable-pressure lattice parameters of cubic CuPt in $Pm\bar{3}m$ collected at I15, Diamond Light Source. Uncertainty is ± 0.1 GPa at all pressures.

p / GPa	a / Å	V / Å^3
1.39	6.063(12)	223(2)
1.44	6.031(10)	219(2)
1.54	5.998(7)	215.8(8)
1.66	5.996(6)	215.6(7)
1.72	5.9756(6)	213.4(6)
1.80	5.984(5)	214.3(6)
1.86	5.947(5)	210.3(5)
1.91	5.935(5)	209.1(5)
1.98	5.918(5)	207.3(5)
2.08	5.891(4)	204.5(4)

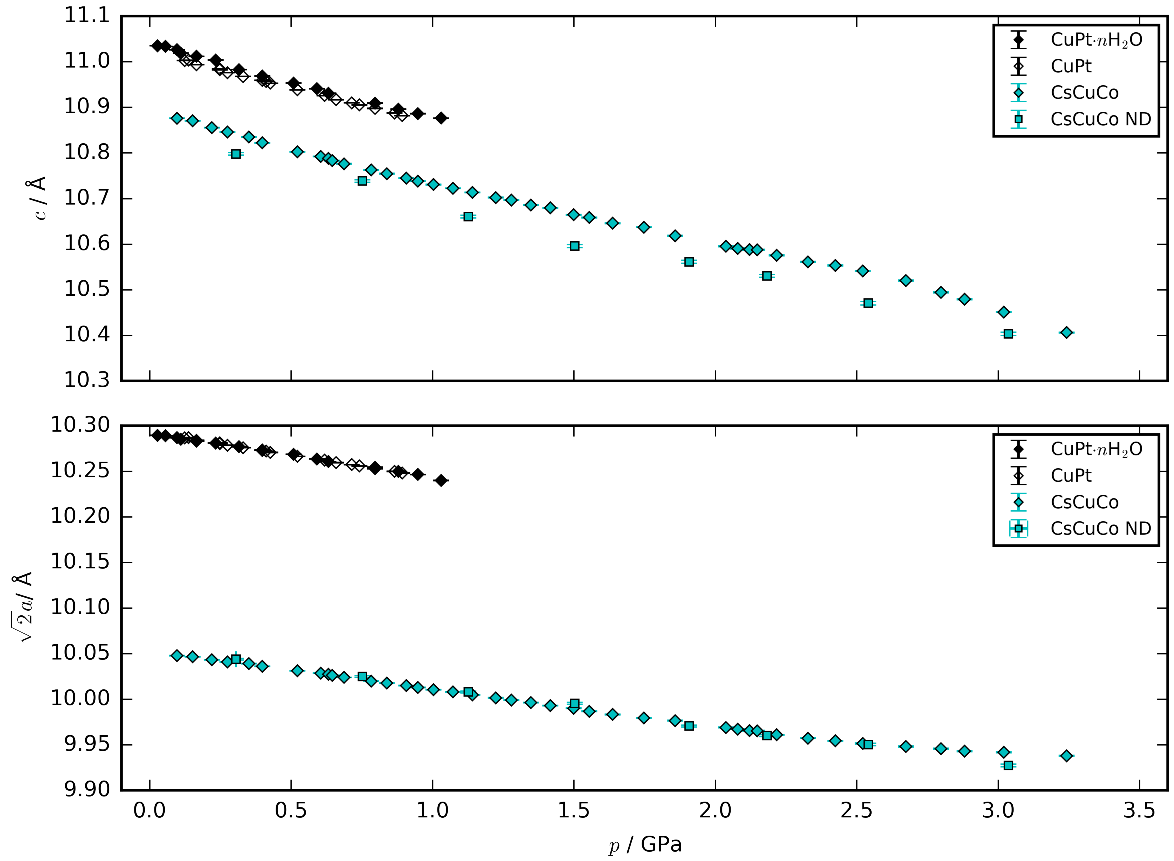

Figure S20: The lattice parameters of CuPt, CuPt·nH$_2$O, and CsCuCo as a function of pressure. All data collected by XRD unless otherwise stated. The uncertainty in the pressure is 0.1 GPa for the XRD data, but horizontal error bars are omitted for clarity.

Table S6: Variable-pressure lattice parameters of CsCuCo in $I\bar{4}m2$ collected at I15, Diamond Light Source. Uncertainty is ± 0.1 GPa at all pressures.

p / GPa	a / Å	c / Å	V / Å^3
0.10	7.1050(3)	10.8761(7)	549.04(5)
0.15	7.1040(2)	10.8706(7)	548.61(5)
0.22	7.1017(3)	10.8555(7)	547.49(5)
0.27	7.1001(3)	10.8463(7)	546.77(5)
0.35	7.0987(3)	10.8352(7)	546.01(5)
0.40	7.0966(3)	10.8223(7)	545.03(5)
0.52	7.0931(2)	10.8027(7)	543.50(5)
0.60	7.0912(2)	10.7923(7)	542.70(5)

Table S6: Variable-pressure lattice parameters of CsCuCo in $I\bar{4}m2$ collected at I15, Diamond Light Source. Uncertainty is ± 0.1 GPa at all pressures.

p / GPa	a / Å	c / Å	V / Å^3
0.63	7.0904(2)	10.7880(7)	542.35(5)
0.65	7.0896(3)	10.7832(7)	541.99(5)
0.69	7.0880(2)	10.7765(7)	541.40(5)
0.78	7.0852(3)	10.7627(7)	540.28(5)
0.84	7.0835(3)	10.7548(7)	539.63(5)
0.91	7.0816(3)	10.7448(8)	538.85(5)
0.95	7.0801(3)	10.7383(8)	538.29(5)
1.00	7.0785(3)	10.7308(8)	537.67(5)
1.07	7.0768(3)	10.7224(8)	536.99(5)
1.14	7.0744(3)	10.7136(8)	536.19(6)
1.22	7.0722(3)	10.7022(9)	535.28(6)
1.28	7.0704(3)	10.6969(9)	534.74(6)
1.35	7.0685(3)	10.6861(9)	533.91(6)
1.42	7.0661(3)	10.6796(9)	533.23(7)
1.50	7.0641(3)	10.6647(9)	532.19(7)
1.55	7.0618(3)	10.6585(9)	531.53(7)
1.64	7.0593(3)	10.6461(10)	530.53(7)
1.75	7.0565(3)	10.6372(10)	529.68(7)
1.86	7.0545(4)	10.6184(10)	528.43(8)
2.04	7.0492(4)	10.5956(11)	526.51(8)
2.08	7.0479(4)	10.5908(11)	526.08(8)
2.12	7.0467(4)	10.5882(11)	525.77(8)
2.15	7.0466(4)	10.5880(11)	525.75(8)

Table S7: Variable-pressure lattice parameters of CsCuCo in $I\bar{4}m2$ collected at PEARL, ISIS Neutron and Muon Source.

p / GPa	a / Å	c / Å	V / Å^3
0.309(8)	7.102(6)	10.798(3)	544.7(2)
0.774(10)	7.089(6)	10.739(3)	539.6(2)

Table S7: Variable-pressure lattice parameters of CsCuCo in $I\bar{4}m2$ collected at PEARL, ISIS Neutron and Muon Source.

p / GPa	a / Å	c / Å	V / Å^3
1.141(10)	7.077(6)	10.661(4)	533.9(3)
1.497(11)	7.068(6)	10.596(4)	529.3(2)
1.899(14)	7.050(7)	10.561(4)	525.0(2)
2.17(2)	7.043(8)	10.530(4)	522.3(3)
2.55(3)	7.036(7)	10.471(5)	518.4(3)
3.04(3)	7.020(7)	10.403(3)	512.6(2)

Table S8: Variable-pressure lattice parameters of RbCuCo in $Cccm$ collected at I15, Diamond Light Source. Uncertainty is ± 0.1 GPa at all pressures.

p / GPa	a / Å	b / Å	c / Å	V / Å^3
0.03	10.8498(7)	9.9749(6)	10.0365(5)	1086.20(11)
0.05	10.8424(7)	9.9723(6)	10.0343(6)	1084.95(11)
0.08	10.8376(7)	9.9715(6)	10.0337(6)	1084.32(11)
0.12	10.8243(6)	9.9675(6)	10.0320(5)	1082.36(10)
0.14	10.8235(6)	9.9673(5)	10.0321(5)	1082.28(10)
0.16	10.8142(7)	9.9640(6)	10.0302(5)	1080.78(10)
0.19	10.8041(7)	9.9612(6)	10.0288(5)	1079.31(10)
0.22	10.7984(7)	9.9591(6)	10.0277(5)	1078.41(10)
0.25	10.7919(7)	9.9573(6)	10.0267(5)	1077.45(10)
0.27	10.7834(7)	9.9544(6)	10.0250(5)	1076.11(10)
0.32	10.7736(7)	9.9517(5)	10.0236(5)	1074.68(10)
0.36	10.7639(7)	9.9489(6)	10.0221(5)	1073.25(11)
0.37	10.7591(7)	9.9474(6)	10.0215(5)	1072.55(11)
0.41	10.7512(8)	9.9449(6)	10.0202(5)	1071.36(11)
0.44	10.7427(8)	9.9423(6)	10.0188(5)	1070.08(11)
0.47	10.7329(9)	9.9396(6)	10.0173(5)	1068.65(12)
0.51	10.7211(9)	9.9360(6)	10.0152(5)	1066.87(12)
0.55	10.7105(10)	9.9331(6)	10.0141(5)	1065.38(13)
0.62	10.6945(12)	9.9283(7)	10.0119(6)	1063.0(2)

Table S8: Variable-pressure lattice parameters of RbCuCo in $Cccm$ collected at I15, Diamond Light Source. Uncertainty is ± 0.1 GPa at all pressures.

p / GPa	a / Å	b / Å	c / Å	V / Å^3
0.67	10.6754(10)	9.9232(6)	10.0092(5)	1060.32(13)
0.77	10.6523(9)	9.9152(6)	10.0053(5)	1056.75(12)
0.80	10.6405(9)	9.9107(6)	10.0036(5)	1054.93(12)
0.85	10.6360(9)	9.9087(6)	10.0027(5)	1054.18(12)
0.88	10.6259(8)	9.9049(6)	10.0008(5)	1052.57(12)
0.92	10.6142(8)	9.9001(6)	9.9987(5)	1050.69(11)
1.00	10.5979(8)	9.8935(6)	9.9959(5)	1048.08(11)
1.09	10.5786(8)	9.8856(6)	9.9926(6)	1044.98(11)
1.18	10.5549(8)	9.8762(7)	9.9889(6)	1041.28(12)
1.29	10.5304(8)	9.8661(7)	9.9850(6)	1037.39(12)
1.36	10.5133(8)	9.8595(7)	9.9824(6)	1034.73(12)
1.42	10.4992(8)	9.8532(7)	9.9800(6)	1032.44(12)
1.51	10.4798(8)	9.8452(7)	9.9771(6)	1029.39(13)
1.61	10.4574(9)	9.8357(8)	9.9735(7)	1025.83(13)
1.69	10.4365(9)	9.8260(8)	9.9703(7)	1022.44(14)
1.79	10.4157(9)	9.8165(8)	9.9672(7)	1019.10(14)
1.89	10.3936(10)	9.8064(8)	9.9634(7)	1015.52(15)
1.97	10.3753(10)	9.7980(9)	9.9606(7)	1012.57(15)
2.05	10.3559(10)	9.7888(9)	9.9573(8)	1009.4(2)
2.13	10.3343(11)	9.7788(10)	9.9529(8)	1005.8(2)

Table S9: Variable-pressure lattice parameters of RbCuCo in $Cccm$ collected at PEARL, ISIS Neutron and Muon Source.

p / GPa	a / Å	b / Å	c / Å	V / Å^3
0.282(4)	10.7926(6)	9.9496(7)	10.0193(6)	1075.91(11)
0.546(7)	10.7261(7)	9.9292(8)	10.0110(6)	1066.18(12)
0.822(8)	10.6676(8)	9.9069(9)	10.0033(7)	1057.17(13)
1.121(10)	10.6034(10)	9.8815(10)	9.9929(8)	1047.0(2)
1.425(11)	10.5392(10)	9.8584(11)	9.9838(10)	1037.3(2)

Table S9: Variable-pressure lattice parameters of RbCuCo in $Cccm$ collected at PEARL, ISIS Neutron and Muon Source.

p / GPa	a / Å	b / Å	c / Å	V / Å^3
1.801(13)	10.4629(12)	9.8222(13)	9.9708(11)	1024.7(3)
2.24(2)	10.3701(15)	9.776(2)	9.9547(14)	1009.2(3)
2.82(3)	10.259(2)	9.727(2)	9.939(2)	991.8(4)
3.57(4)	10.160(3)	9.663(3)	9.913(3)	973.2(5)
4.25(5)	10.071(4)	9.595(4)	9.885(4)	955.2(6)
4.82(7)	10.001(4)	9.554(5)	9.851(4)	941.3(7)

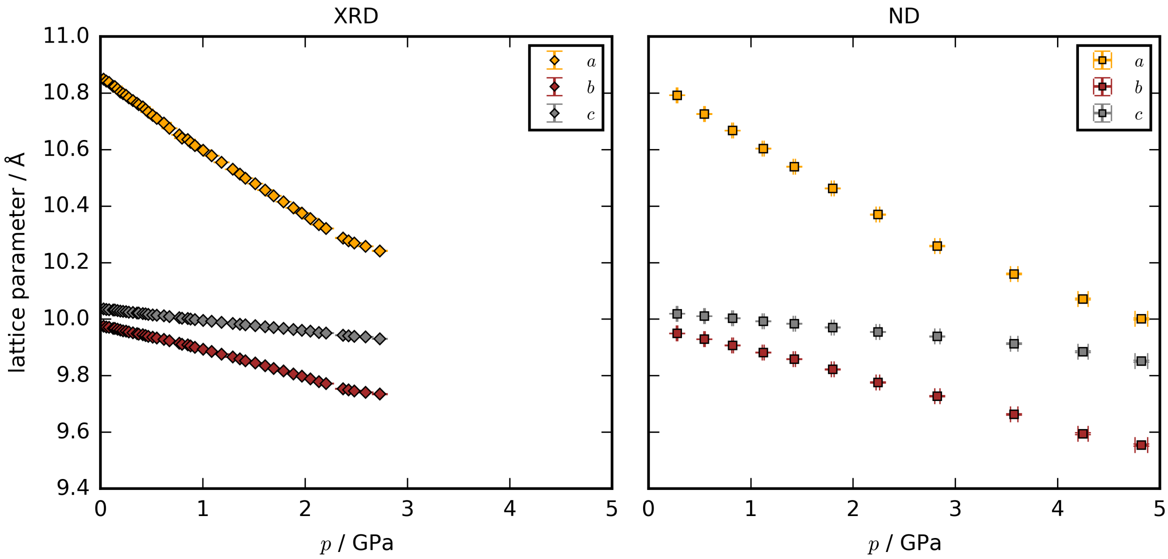

Figure S21: The lattice parameters of RbCuCo as a function of pressure. The uncertainty in the pressure is 0.1 GPa for the XRD data, but horizontal error bars are omitted for clarity.

5 Birch–Murnaghan fits

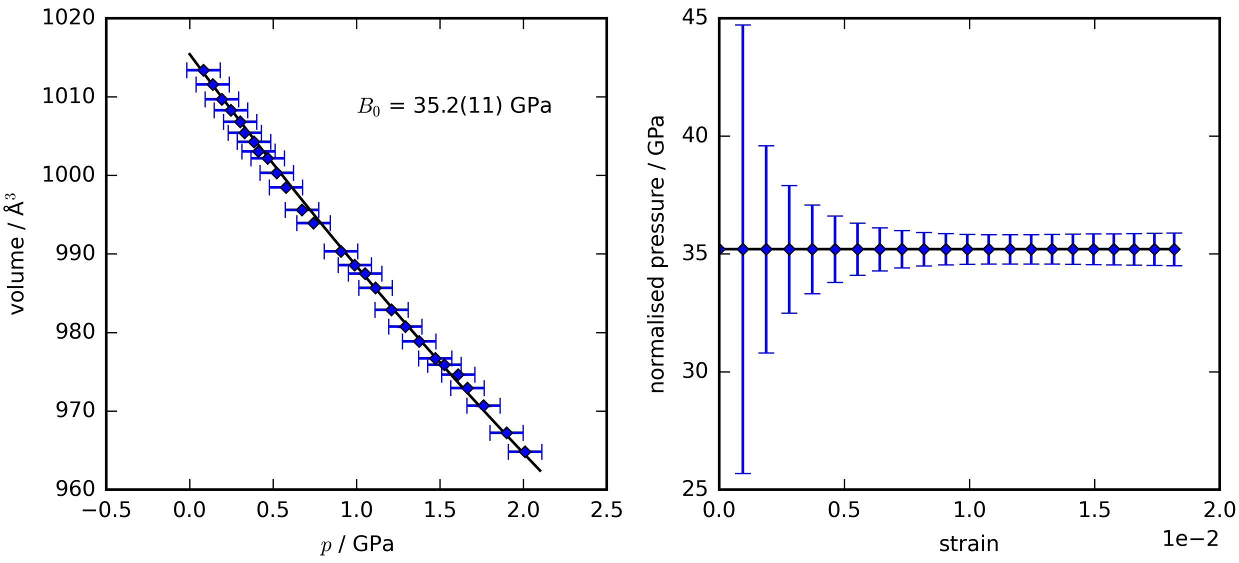

Figure S22: Fits to experimental unit cell volumes using second-order Birch–Murnaghan equation of states and the normalised pressure *vs.* Eulerian strain for Cu[Co]$_{0.67} \cdot n$H$_2$O, using data collected at I15, Diamond Light Source.

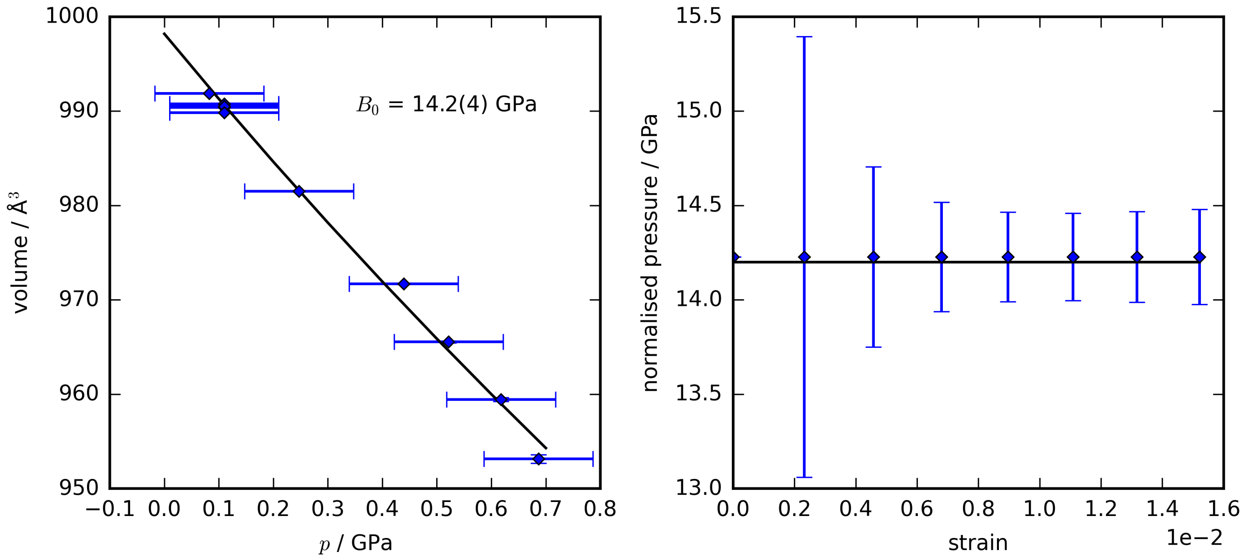

Figure S23: Fits to experimental unit cell volumes using second-order Birch-Murnaghan equation of states and the normalised pressure *vs.* Eulerian strain for Cu[Co]$_{0.67}$, using data collected at I15, Diamond Light Source.

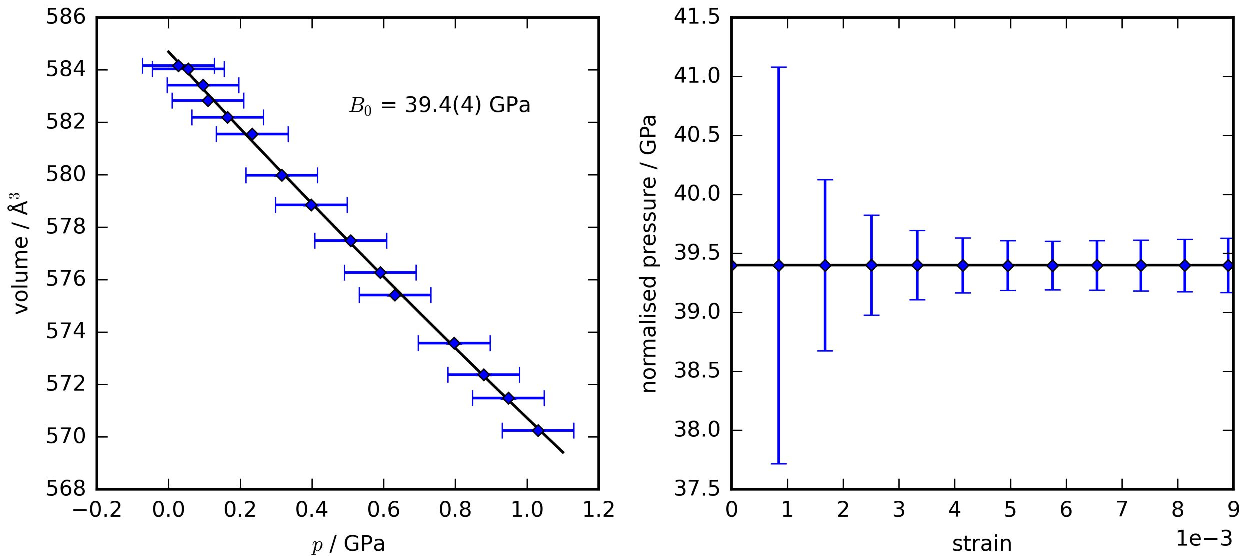

Figure S24: Fits to experimental unit cell volumes using second-order Birch–Murnaghan equation of states and the normalised pressure *vs.* Eulerian strain for CuPt·nH$_2$O, using data collected at I15, Diamond Light Source.

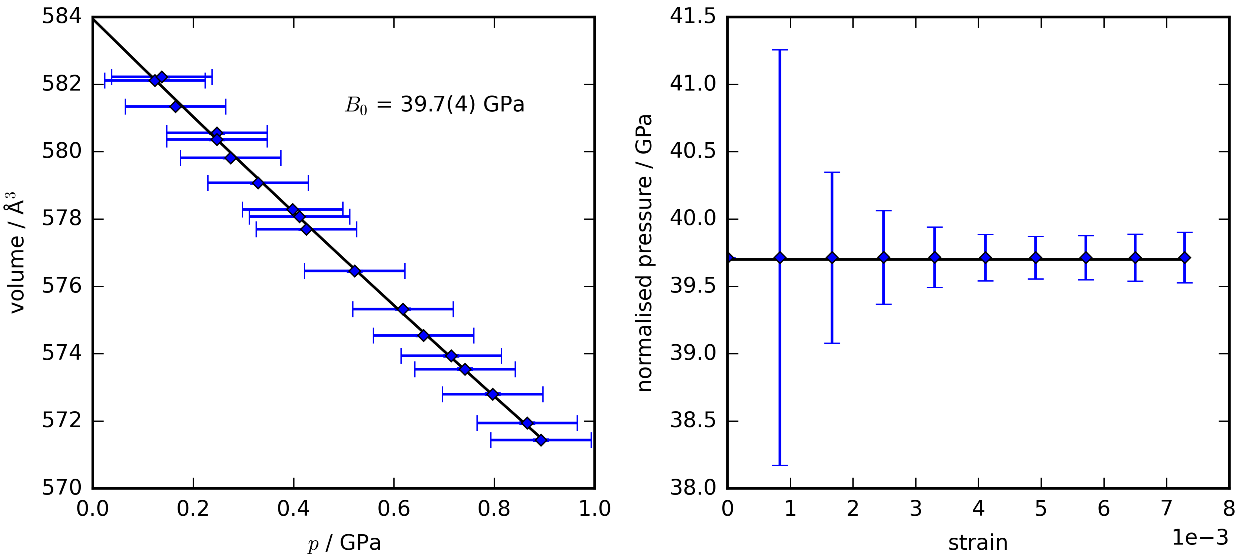

Figure S25: Fits to experimental unit cell volumes using second-order Birch–Murnaghan equation of states and the normalised pressure *vs.* Eulerian strain for CuPt, using data collected at I15, Diamond Light Source.

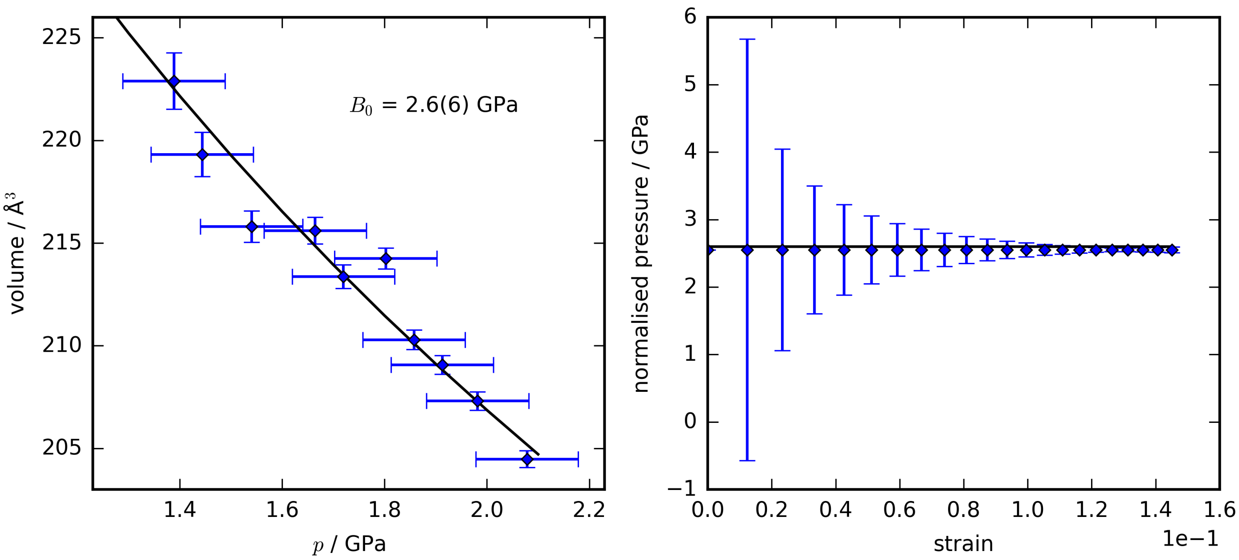

Figure S26: Fits to experimental unit cell volumes using second-order Birch–Murnaghan equation of states and the normalised pressure *vs.* Eulerian strain for the cubic phase of CuPt, using data collected at I15, Diamond Light Source.

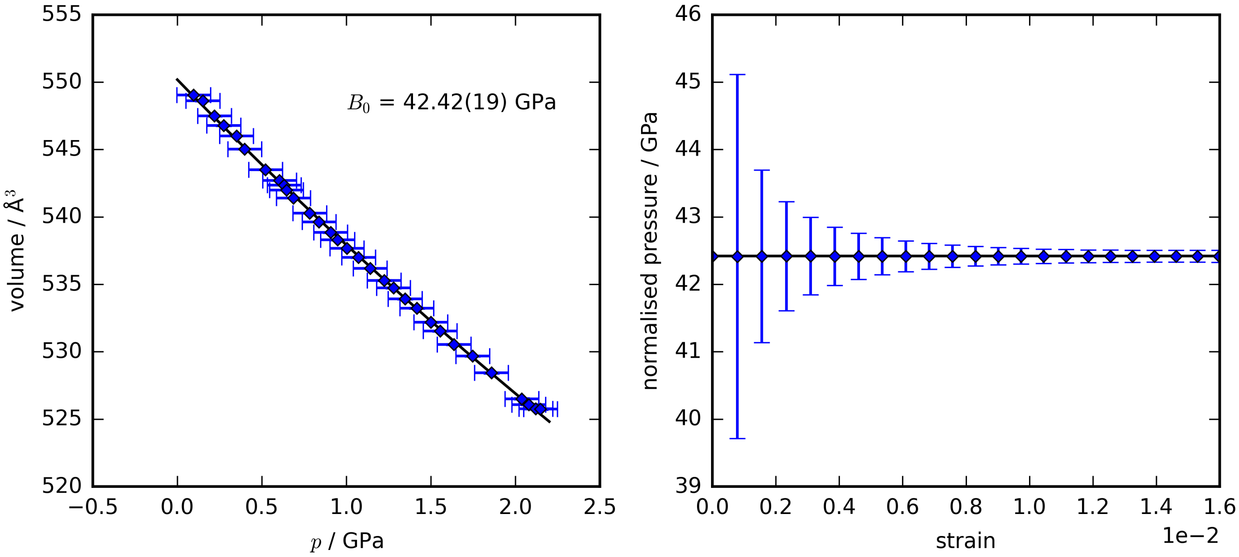

Figure S27: Fits to experimental unit cell volumes using second-order Birch–Murnaghan equation of states and the normalised pressure *vs.* Eulerian strain for CsCuCo, using data collected at I15, Diamond Light Source.

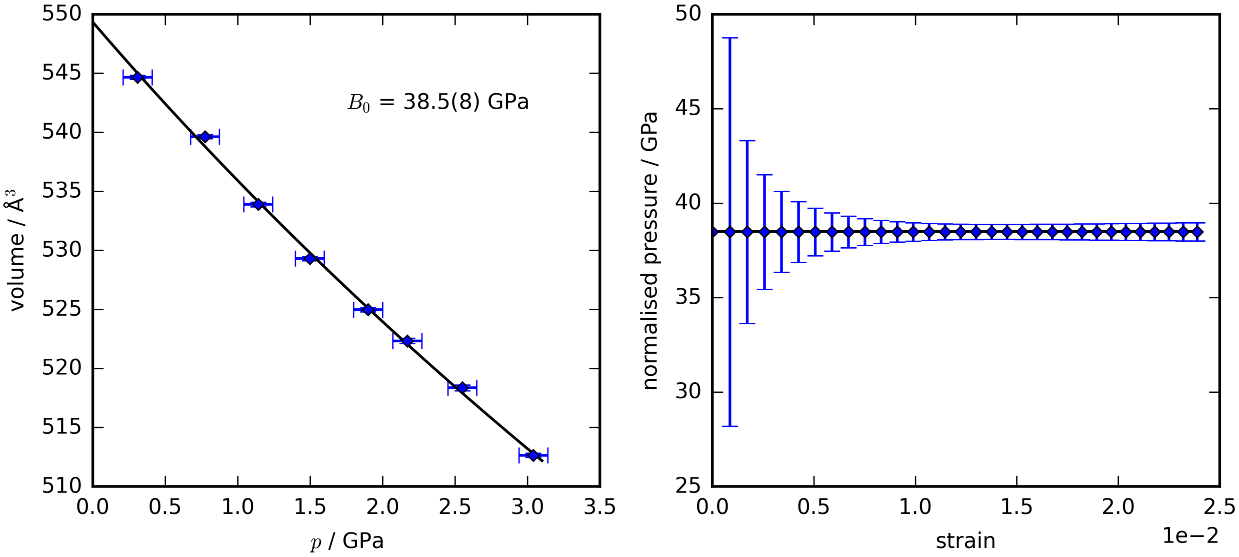

Figure S28: Fits to experimental unit cell volumes using second-order Birch–Murnaghan equation of states and the normalised pressure *vs.* Eulerian strain for CsCuCo, using data collected at PEARL, ISIS Neutron and Muon Source.

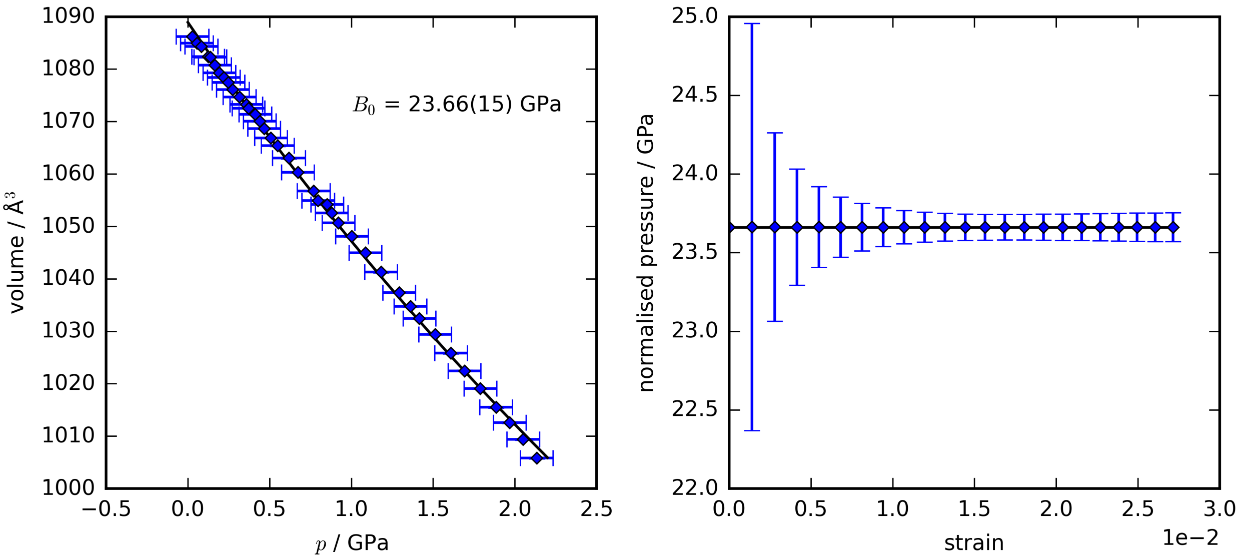

Figure S29: Fits to experimental unit cell volumes using second-order Birch–Murnaghan equation of states and the normalised pressure *vs.* Eulerian strain for RbCuCo, using data collected at I15, Diamond Light Source.

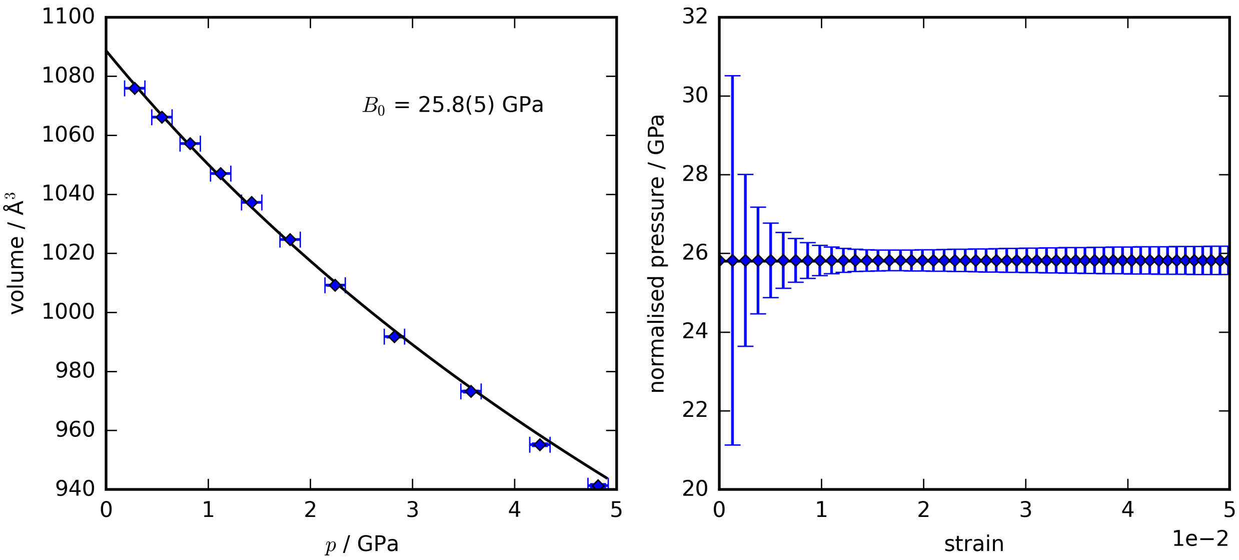

Figure S30: Fits to experimental unit cell volumes using second-order Birch–Murnaghan equation of states and the normalised pressure *vs.* Eulerian strain for RbCuCo, using data collected at PEARL, ISIS Neutron and Muon Source.

6 References

(S1) H. L. B. Boström and R. I. Smith, *Chem. Commun.*, 2019, **55**, 10230–10233.

(S2) C. Prescher and V. B. Prakapenka, *High Pressure Res.*, 2015, **35**, 223–230.

(S3) C. L. Bull, N. P. Funnell, M. G. Tucker, S. Hull, D. J. Francis and W. G. Marshall, *High Pressure Res.*, 2016, **36**, 493–511.

(S4) O. Arnold, J. C. Bilheux, J. M. Borreguero, A. Buts, S. I. Campbell, L. Chapon, M. Doucet, N. Draper, R. Ferraz Leal, M. A. Gigg, V. E. Lynch, A. Markvardsen, D. J. Mikkelson, R. L. Mikkelson, R. Miller, K. Palmen, P. Parker, G. Passos, T. G. Perring, P. F. Peterson, S. Ren, M. A. Reuter, A. T. Savici, J. W. Taylor, R. J. Taylor, R. Tolchenov, W. Zhou and J. Zikovsky, *Nucl. Instrum. Methods Phys. Res., Sect. A*, 2014, **764**, 156–166.

(S5) A. A. Coelho, *J. Appl. Cryst.*, 2018, **51**, 210–218.

(S6) G. S. Pawley, *J. Appl. Cryst.*, 1981, **14**, 357–361.

(S7) H. M. Rietveld, *Acta Crystallogr.*, 1967, **22**, 151–152.

(S8) B. J. Campbell, H. T. Stokes, D. E. Tanner and D. M. Hatch, *J. Appl. Cryst.*, 2006, **39**, 607–614.

(S9) H. J. Buser, D. Schwarzenbach, W. Petter and A. Ludi, *Inorg. Chem.*, 1977, **16**, 2704–2710.

(S10) F. Birch, *Phys. Rev.*, 1947, **71**, 809–824.

(S11) F. D. Murnaghan, *Proc. Natl. Acad. Sci. U.S.A*, 1944, **30**, 244–247.

(S12) R. J. Angel, J. Gonzalez-Platas and M. Alvaro, *Z. Kristallogr.*, 2014, **229**, 405–419.

(S13) K. Yokogawa, K. Murata, H. Yoshino and S. Aoyama, *Jpn. J. Appl. Phys.*, 2007, **46**, 3636—3639.

(S14) M. J. Cliffe and A. L. Goodwin, *J. Appl. Cryst.*, 2012, **45**, 1321–1329.
